# Histone 4 lysine 8 acetylation regulates proliferation and host–pathogen interaction in *Plasmodium falciparum*

**DOI:** 10.1186/s13072-017-0147-z

**Published:** 2017-08-22

**Authors:** Archana P. Gupta, Lei Zhu, Jaishree Tripathi, Michal Kucharski, Alok Patra, Zbynek Bozdech

**Affiliations:** School of Biological Sciences, Nanyang Technological University, 60 Nanyang Drive, Singapore, 637551 Singapore

**Keywords:** *P. falciparum*, H4K8ac, Chromatin, Transcriptional dynamics, HDAC inhibitors, ChIP-Seq

## Abstract

**Background:**

The dynamics of histone modifications in *Plasmodium falciparum* indicates the existence of unique mechanisms that link epigenetic factors with transcription. Here, we studied the impact of acetylated histone code on transcriptional regulation during the intraerythrocytic developmental cycle (IDC) of *P. falciparum*.

**Results:**

Using a dominant-negative transgenic approach, we showed that acetylations of histone H4 play a direct role in transcription. Specifically, these histone modifications mediate an inverse transcriptional relationship between the factors of cell proliferation and host–parasite interaction. Out of the four H4 acetylations, H4K8ac is likely the rate-limiting, regulatory step, which modulates the overall dynamics of H4 posttranslational modifications. H4K8ac exhibits maximum responsiveness to HDAC inhibitors and has a highly dynamic distribution pattern along the genome of *P. falciparum* during the IDC. Moreover, H4K8ac functions mainly in the euchromatin where its occupancy shifts from intergenic regions located upstream of 5′ end of open reading frame into the protein coding regions. This shift is directly or indirectly associated with transcriptional activities at the corresponding genes. H4K8ac is also active in the heterochromatin where it stimulates expression of the main antigenic gene family (*var*) by its presence in the promoter region.

**Conclusions:**

Overall, we demonstrate that H4K8ac is a potential major regulator of chromatin-linked transcriptional changes during *P. falciparum* life cycle which is associated not only with euchromatin but also with heterochromatin environment. This is potentially a highly significant finding that suggests a regulatory connection between growth and parasite–host interaction both of which play a major role in malaria parasite virulence.

**Electronic supplementary material:**

The online version of this article (doi:10.1186/s13072-017-0147-z) contains supplementary material, which is available to authorized users.

## Background

*Plasmodium falciparum*, one of the protozoan parasites responsible for malaria in humans, exhibits coordinated mechanisms of transcriptional regulation during the development through its life cycle. There is mounting evidence that epigenetic mechanisms contribute to this unique gene regulation and thus are vital for the parasite’s growth and development [1–10]. Besides, absence of the linker histone H1 [11], scarcity of transcription factors [12] and lack of a functional RNA interference system [13] has led to a working model in which histone posttranslational modifications (PTMs) play a pivotal role in *P. falciparum* gene regulation. Based on this model, *Plasmodium* epigenome is considered mainly euchromatic [14–16], marked by unique combinations of histone variants and their posttranslational modifications (PTMs) [11, 17–19]. The abundance of parasite specific histone modifications is suggestive of the role of epigenetic mechanisms regulating parasite virulence [18, 19]. Intriguingly, the genome-wide distribution of histone variants and their PTMs is highly dynamic across the *P. falciparum* intraerythrocytic developmental cycle (IDC) [20–23]. Like mRNA abundance, occupancy of many histone marks such as H4K8ac and H3K9ac shows single peak profiles across the IDC in a large proportion of the *P. falciparum* genes [21]. On the other hand, there are histone PTMs whose occupancy is constant throughout the IDC. These include canonical heterochromatin markers such as H3K9me3 and H3K36me3, implicated in gene silencing [14, 24, 25] and few euchromatin histone marks such as H4K5ac and H3K14ac, either abundantly spread through the majority of the genome or confined to a small number of genomic loci [21]. Occupancy of histone variants also contributes to the dynamic chromatin remodeling throughout the *P. falciparum* IDC with some variants which associate with actively transcribed genes, while others play roles in chromatin structure [20, 26–28]. Chromatin remodeling is also affected by dynamic nucleosome structures [29–33] and chromatin binding proteins [34–37] in *P. falciparum.* Likewise, the dynamics is reflected in occupancy of RNA polymerase II exhibiting distinct patterns for early and late expressed genes [38]. Altogether these findings suggest that the time component of the occupancy profiles across the IDC is one of the variables of the overall “histone code” playing a key fundamental role in gene expression during the *Plasmodium* life cycle.

Studying 13 canonical PTMs of H4 and H3, we have previously shown that acetylation of H4 at lysine residue 8 (H4K8ac) is among the most dynamic modifications occupying predominantly the 5′ intergenic regions (5′IGRs) and 5′ termini of the open reading frames (ORFs) of more than half of the *P. falciparum* genes [21]. The single peak occupancy profiles of the 5′IGR/ORF-bound H4K8ac showed good correlation with the respective mRNA profiles across the IDC. This was highly surprising given that the “neighboring” PTMs at the H4 “tail” (H4K5ac and H4K12ac) exhibited a highly abundant but constant occupancy throughout the vast majority of the genome. Treatment of *P. falciparum* parasites with the Class I and II histone deacetylase (HDAC) inhibitor apicidin resulted in induction of the overall protein levels of H4K8ac as well as its occupancy across the genome [39]. H4K8ac (together with H3K9ac) was also found to be an effector of the DNA damage stress response, being induced by treatment of *P. falciparum* parasites with methyl methanesulfonate (MMS) at multiple stages of the IDC [40]. The MMS-induced levels of H4K8ac in *P. falciparum* coincide with transcriptional induction of stress responses. Intriguingly, artemisinin, the main chemotherapeutics for malaria treatment, solicited a similar effect characterized by increased levels of H4K8ac and upregulation of the stress response genes [40]. This strongly suggests that H4K8ac plays a role in transcription regulation associated with both, progression of the *Plasmodium* life cycle, and, responses to external perturbations/stresses. Surprisingly, in other eukaryotic organisms, H4K8ac is yet to be implicated in any major processes of epigenetic regulation of gene expression. In yeast and humans, H4K8ac seems to play an auxiliary role in transcription, being a part of an overall euchromatin-linked histone PTM complex that occupies active promoters [41, 42]. This may suggest that in contrast to most eukaryotes, during the *Plasmodium* evolution, H4K8ac acquired new functions in epigenetic regulation of gene expression and possibly emerged as one of the most crucial histone marks.

All above-mentioned studies, however, provided only associative evidence of H4K8ac involvement in transcription showing modest albeit statistically significant overlaps between H4K8ac-bound genetic loci and transcriptionally deregulated genes [21, 39, 40]. Here, we wanted to establish direct links between H4K8ac and transcription, and evaluate these in context of the neighboring H4 PTMs (H4K5ac, H4K12ac and H4K16ac) detected in *P. falciparum*. For this, we suppressed the studied acetylations in *P. falciparum* cell lines that (over) expressed H4 genes with mutations at lysines 5, 8, 12, 16. The hypoacetylation of histone H4 resulted in broad transcriptional changes characterized by upregulation of genes involved in cell proliferation and downregulation of genes of host–parasite interactions and antigenic presentation/variation. Accordingly, there was an increase in multiplication rate of *P. falciparum* parasites when H4K8ac or all 4 H4 acetylations were suppressed. On the other hand, H4K8 exhibited maximum level of hyperacetylation induced by HDAC inhibitors compared to all other euchromatin marks; however, this hyperacetylation is highly transient. ChIP-Seq results showed that H4K8ac shifts its position back-and-forth between the 5′IGRs in the early and late IDC stages to the 5′ORF in the middle IDC stage. Besides its euchromatin-linked roles, H4K8ac also functions in the context of heterochromatin where it is involved in activation of the major antigenic variation gene family (*var*).

## Results

### Dominant-negative transgenic lines for H4 acetylations

**Fig. 1:**
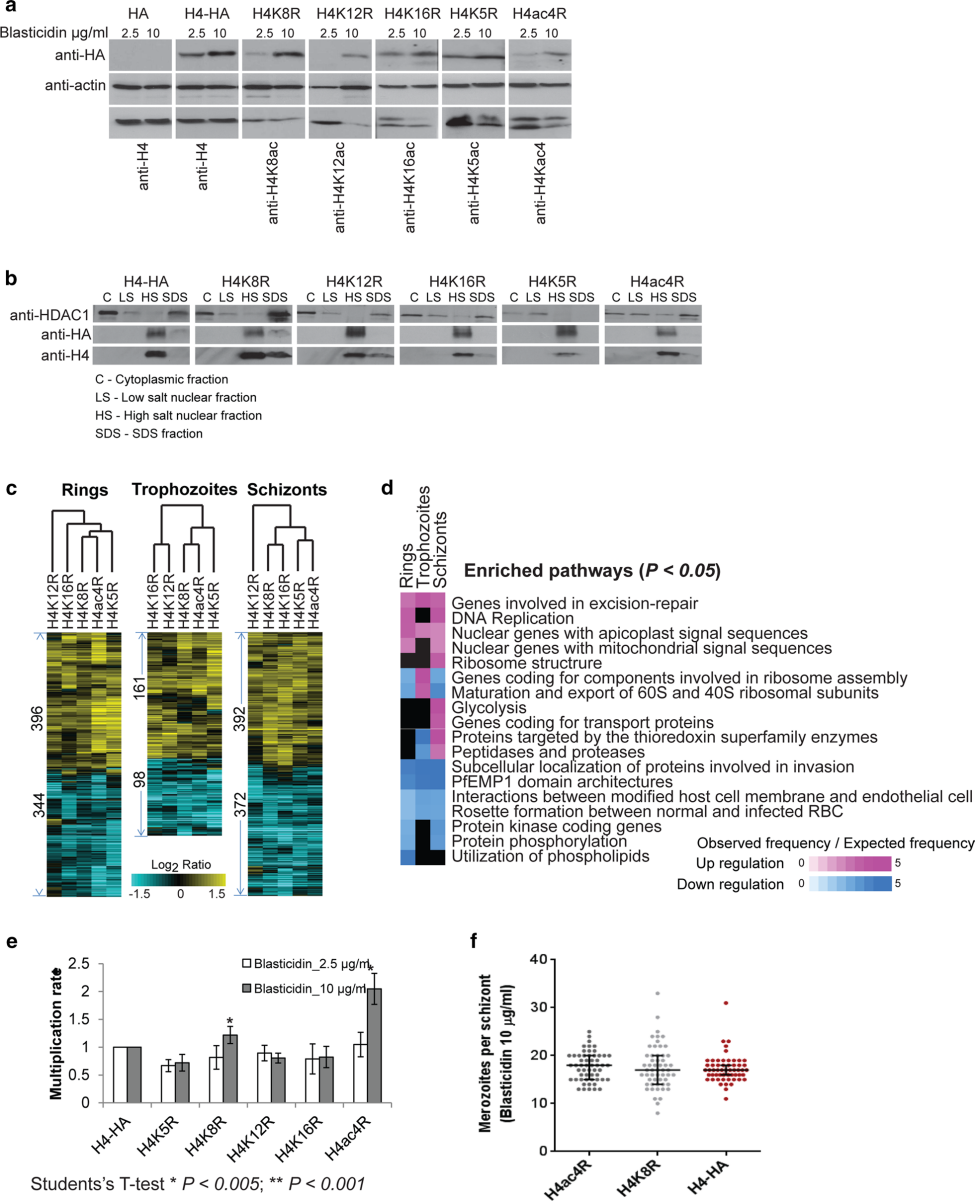
Histone H4 acetylation mutants. **a** Western blot analysis using total protein from the transfectants grown at 2.5 and 10 µg/ml blasticidin to check the presence of HA-tagged H4 proteins using antibodies against HA. Histone antibodies were used as positive control to confirm the expression of endogenous histones and actin was used as a loading control. **b** Western blot analysis carried out from cytoplasmic, low salt nuclear, high salt nuclear and total SDS fractions to confirm nuclear localization of HA-tagged H4 proteins in the transfectants. **c** Microarray expression analysis for transfectants grown at 10 µg/ml blasticidin. *Heat maps* represent the differentially expressed genes in the transfectants at each stage (Student’s *t* test, *P* < 0.05). Data shown is log_2_ ratio of average of biological triplicates, normalized to H4-HA transfectant. **d** Functional pathways significantly up or downregulated in the differentially expressed gene sets (data from **c**) of the transfectants. The *scale* shows the fold enrichment of number of genes in the specific pathways [based on hypergeometric test (*P* < 0.05)]. **e** Effect of H4 mutations on multiplication. Cells were grown in 2.5 or 10 µg/ml blasticidin and FACS using Hoechst stain was carried out to count the number of new rings at each invasion cycle for 6 cycles. **f** The scatter plot of merozoite number in the segmented schizonts in H4ac4R, H4K8R and H4-HA transfectants determined by Giemsa stain light microscopy

In the first step, we created dominant-negative *P*. *falciparum* transgenic parasite lines transfected with pBcamR plasmid containing HA-tagged H4 genes in which the targeted lysine (K) residues (K5, K8, K12, K16) where replaced with arginine (R) (Additional file 1). In total, we generated five transgenic lines with the individual mutations (H4K5R, H4K8R, H4K12R, H4K16R) or with all 4 lysines changed to arginine (H4ac4R) and two additional control lines with either HA-tag only (HA) or wild-type HA-tagged H4 gene (H4-HA). As expected, there was an increase in the plasmid copy number and expression of the transgenic H4 proteins in *P. falciparum* grown in 10 μg/ml of blasticidin compared to 2.5 μg/ml in all parasite lines (Additional file 2: Figure S1a and Fig. 1a, respectively). Cell lysate fractionation showed that the transgenic H4 proteins could be solubilized only by high salt extraction of the nuclear fraction (Fig. 1b). More importantly, the intensities of HA-tagged H4 proteins were found to be, 3.12-fold higher for H4K8R, 2.6-fold higher for H4K12R and 3.6-fold higher for H4K16R compared to endogenous H4 protein in the transfectants at 10 μg/ml of blasticidin (Fig. 1a and Additional file 2: Figure S1h). This indicates partial but significantly higher displacement of endogenous proteins by the HA-tagged H4 protein. Transgenic proteins were also detected in the acid-extracted histone fractions and immuno-fluorescence microscopy of H4 K8R confirmed its nuclear localization (Additional file 2: Figure S1b). Taken together, these results demonstrate that the episomally expressed H4 proteins are targeted to the *P. falciparum* nucleosomes, presumably displacing the endogenous H4 protein and by that suppressing the respective acetylations. Hence, these transgenic lines provide an experimental tool to investigate the role of H4 lysine residues in epigenetic regulation of gene expression during *P. falciparum* IDC.

### Functional implication of the dominant-negative inhibition of H4

Next, we carried out transcriptome analyses of the transfectants grown in the presence of 10 μg/ml of blasticidin. By comparing the transcriptional profiles of the H4 K/R mutants with those from both controls at three stages of the IDC, we observed broad transcriptional changes (Fig. 1c). Remarkably, there appears to be strong similarities between the transcriptional changes induced by the dominant-negative effect of all five H4K/R mutants. Overall, there were higher number of genes that were differentially expressed in the ring and schizont compared to the trophozoite stages. The transcription patterns of the dominant-negative transfectants was not or negatively correlated to HA and positively correlated to each other when normalized to H4-HA (Additional file 2: Figure S1c). On the other hand, there were essentially no transcriptional changes between the unmutated H4-HA and transfectant with the empty vector (HA) also grown at 10 μg/ml of blasticidin (Additional file 2: Figure S1d). Altogether, these results indicate that the acetylations at the 4 lysine residues of H4 act in a similar way possibly reflecting a synergistic and/or complimentary function in transcription during the *P. falciparum* IDC. Nonetheless, there were subtle differences within the individual profiles with H4ac4R clustering closer with H4K8R in the ring and trophozoite stages (Pearson’s correlation coefficient 0.75 and 0.71, respectively) compared to the other 3 mutants (Fig. 1c; Additional file 2: Figure S1c). Given that H4ac4R shows consistently the strongest effect on transcription, this may suggest a key role of H4K8ac in this process.

Functional analyses of the induced transcriptional changes showed a high consistency in the altered mRNA levels for all enriched pathways for all four H4 acetylations (Fig. 1d; Additional file 3: Table S1, Additional file 4: Figure S2). Moreover, similar pathways were deregulated in the H4K8R transfectant at the schizont stage when the blasticidin concentration was increased from 2.5 to 10 μg/ml whereas H4-HA grown at 2.5 and 10 µg/ml of blasticidin induced very few transcriptional changes (Additional file 2: Figure S1e), confirming that the transcriptional deregulation is a direct result of dominant-negative expression of the mutated H4 protein. Namely, the overexpression of the H4 mutants caused upregulation of a wide range of genes involved in cellular pathways associated with growth and replication (e.g., proliferation) of *P. falciparum* during the IDC. These include *genes encoding factors of the assembly of cytoplasmic and the organellar ribosomes*, *glycolysis*, *DNA replication* and *nuclear encoded genes for apicoplast and mitochondrial protein transport* (annotated by MPMP; http://mpmp.huji.ac.il/) (Fig. 1d). In addition, there was an upregulation of two classes of stress response pathways including the *DNA repair machinery* and *thioredoxin metabolism*. While some pathways (such as DNA repair) were upregulated throughout the IDC, others were typically de-repressed during their transcriptional downturn. For example, the genes coding for 40S, 50S and 60S cytoplasmic ribosomal proteins normally expressed during rings [43] were upregulated in the trophozoite and schizont stages, whereas genes encoding for mitochondrial and apicoplast ribosomal proteins normally expressed in the schizonts were upregulated in the rings (Additional file 2: Figure S1f). This indicates that H4 acetylations facilitate transcriptional suppression of proliferation related genes in a life cycle-specific manner, hence regulating the growth of parasite. This occurs in all three major developmental stages of the IDC in which the transgenic histones were expressed at the protein levels (Additional file 2: Figure S1g). Interestingly, the disruptions of H4K8ac or the 4 acetylations lead to significant increases in the parasite multiplication rate (MR) by up to 1.3-fold and twofold, respectively (Fig. 1e). This is most likely a direct result of the induced levels of the mutated H4 proteins as no alterations in MR were observed at 2.5 μg/ml of blasticidin in all transfectant parasites lines. Surprisingly, this was not associated with an increased number of newly formed merozoites in the segmented schizonts measured by light microscopy (Fig. 1f). Moreover, both the mutant and non-mutant transfectants have an identical length of the IDC, measured by transcriptomics analysis (Additional file 5: Table S5). This suggests that the increased MR is likely facilitated by a higher fraction of viable merozoite with elevated levels of infectivity. It will be interesting to explore this possibility in future studies.

The most predominant functional groups enriched among the downregulated genes were factors of host–parasite interactions annotated as *PfEMP1 domain architecture*, *rosette formation between normal and infected RBC* and *interactions between modified host cell membrane and endothelial cells* (Fig. 1d). In particular, we observed downregulation of essentially all members of two gene families implicated in antigenic variation and immune evasion, *var* and *rifin*. The other important downregulated pathway consisted of genes involved in *merozoite invasion*, which includes merozoite surface proteins, rhoptry associated proteins, reticulocyte binding protein homologs and erythrocyte binding antigens (Additional file 3: Table S1). In contrast, downregulation of these genes was independent of the developmental stage, typically occurring throughout the IDC (Fig. 1d; Additional file 4: Figure S2). We validated that the 40S and 50S cytoplasmic ribosomal proteins were upregulated by, 1.7 to 2-fold, and, 1.7- to 4.3-fold, in trophozoite and schizont stages, respectively. On the other hand, invasion-related genes, RAP2 and RAP3, and, MSP and RAP3, were confirmed to be downregulated showing a fold change of <1 compared to wild-type parasites in trophozoite and schizont stages, respectively (Additional file 2: Figure S1i). Taken together, these results suggest that the 4 lysine residues of the H4 tail play a central role in regulating several key biological functionalities by which the parasite adapts to its environment. Specifically, the H4 acetylations facilitate an inverse regulatory relationship between growth and multiplication on one-side and host–parasite interactions on the other.

### H4K8ac is the dynamic component of the H4 tetra-acetylation moiety

**Fig. 2:**
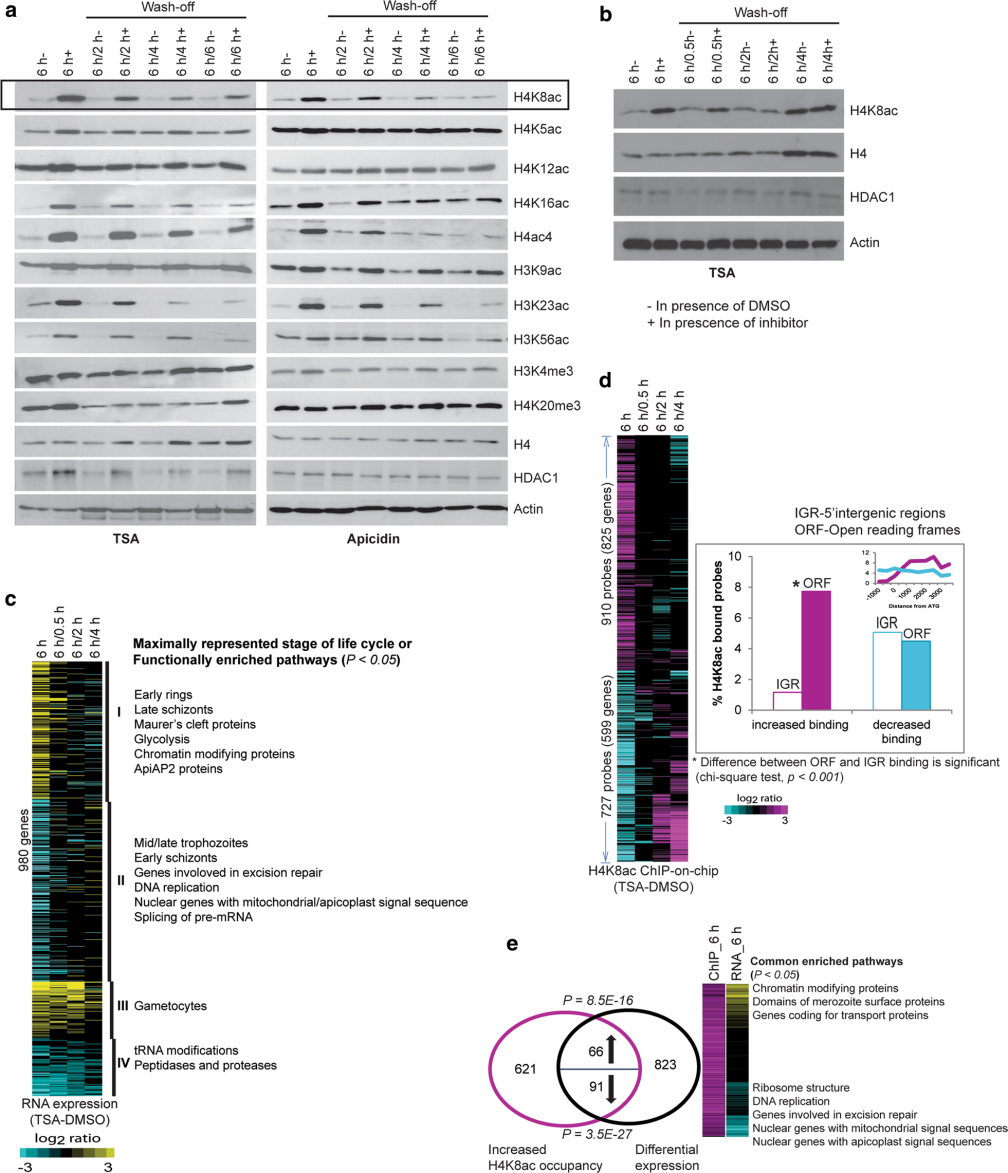
Effect of HDAC inhibitors on *P. falciparum*. **a** Western blot analysis to check the effect of HDAC inhibitors on histone modifications. Trophozoite stage parasites were cultured for 6 h with DMSO (6 h−), 70 nM of Trichostatin A (TSA) or 75 nM of Apicidin (6 h+). The drug was subsequently washed off, and the cells were grown for another 2 h (6 h/2 h), 4 h (6 h/4 h) or 6 h (6 h/6 h). The antibodies used for each blot are shown on the right. **b** Similar experiment was repeated with TSA treatment for 6 h, subsequent wash off and growth for 0.5 h (6 h/0.5 h), 2 h (6 h/2 h) and 4 h (6 h/4 h). Total protein was extracted from the respective samples for western blot analysis using antibodies against histone H4 or H4K8ac or Pf HDAC1. **c** Microarray expression analysis of TSA treated cells. TSA or DMSO treatment for 6 h was done in biological triplicates and subsequent wash-off experiments were done in duplicates. The *heat map* (*left panel*) shows average log_2_ ratios of replicates (TSA minus DMSO) of the genes differentially expressed after 6 h TSA exposure (Student’s *t* test, *P* < 0.05). *Right panel* represents the parasite stage whose genes are maximally expressed as well as the functionally enriched MPM pathways in each of the clusters. **d** ChIP-on-chip using H4K8ac antibody to assess differential binding upon TSA treatment. All experiments were done in at least duplicates. The *heat map* on the *left* shows average log_2_ ratios of replicates (TSA minus DMSO) of the probes differentially acetylated after 6-h TSA exposure (Wilcoxon rank sum test, *P* < 0.05). *Graph* on the *right* shows the percentage of probes that are differentially acetylated upon TSA treatment for 6 h. *Inset graph* shows the differential occupancy across the gene body. **e** *Venn diagram* represents the overlap between differential expression and increased H4K8 occupancy in the ORFs due to TSA. Binomial distribution was used to assign *P* values for up or downregulated genes overlapping with H4K8ac hyperacetylation. *Heat map* on the *right* represents the fold change in H4K8ac occupancy or RNA expression of the hyperacetylated genes. Functional pathways (MPM) significantly enriched in the up- or downregulated gene sets are shown

Next, we wished to compare the responsiveness of the four H4 lysine acetylations to HDAC inhibition in a broader context of *P. falciparum* chromatin remodeling [21, 39, 40]. Highly synchronized trophozoites were treated with IC_90_ concentrations of Trichostatin A (TSA) or apicidin (50 and 70 nM, respectively) for 6 h followed by removal of the drug and culturing for another 2, 4 and 6 h, similar to our previous study [44]. Out of the 4 H4 acetylations, H4K8ac and (to a lesser degree) H4K16ac responded to both HDAC inhibitors, while H4K5 and H4K12 acetylation levels were unchanged (Fig. 2a). Both HDAC inhibitors induced acetylation at other euchromatin marks including H3K23ac, H3K56ac and to lesser degree H3K9ac whereas euchromatin-linked (tri)methylations of H3K4 and H4K20 were unresponsive. Finally, there was a dramatic increase in the signal intensity using an antibody against H4 tetra-acetylation (H4ac4). This likely corresponds to the changes mainly at H4K8ac and (less so) H4K16ac, given that the levels of H4K5ac and H4K12ac appear to be constitutive. As mentioned above, H4K8ac is the most dynamic euchromatin mark whose occupancy is tightly correlated with transcriptional activity, while H4K16ac showed only moderate-to-low levels of occupancy changes and is virtually uncoupled from transcription [21]. This suggests that out of the two dynamic H4 acetylations, H4K8ac may play a pivotal role in regulation of gene expression that is ultimately mediated by the H4ac4 epigenetic moiety. Hence, in the following parts of our study, we focus on H4K8ac as a major regulatory factor in gene expression during the *P. falciparum* IDC.

The effect of both TSA and apicidin on all the responsive histone acetylations appears to be transient as most of the histone modifications returned to their basal levels over 6 h after the compound removal (Fig. 2a). This is consistent with our previous study showing a transient effect of HDAC inhibitors on *P. falciparum* transcriptome [44]. To explore this phenomenon further, we carried out a “treatment/wash-off” time course experiment where the highly synchronized trophozoites were first exposed to 50 nM of TSA and subsequently samples were collected 0, 0.5, 2 and 4 h after the drug removal (Fig. 2b). These samples were used for transcriptomic and ChIP-on-chip analysis of H4K8ac occupancy, simultaneously. In this experimental setting, TSA caused a dramatic deregulation of the IDC transcriptional cascade with a broad downregulation of trophozoite-specific genes and upregulation of genes normally expressed in other stages (early ring and early schizonts) (Fig. 2c). This deregulation is largely reversible such that even 30 min after the drug removal, the majority of the transcripts returned to their original levels. However, there were two gene clusters whose TSA-induced transcriptional change was stable for at least 2 h. These include genes of gametocyte and merozoite stages among the upregulated and *tRNA modifications* and *peptidases and proteases* among the downregulated genes (Fig. 2c; Additional file 6: Table S2). In future studies, it will be interesting to understand the molecular mechanisms that underline both the reversible and irreversible transcriptional changes induced by the HDAC inhibitors. The chromosomal occupancy of H4K8ac appeared also highly sensitive to TSA at least 1637 genetic loci corresponding to 1424 genes (Fig. 2d). There is a remarkable asymmetry in the distribution of the TSA-induced H4K8ac occupancy that is significantly enriched within the ORFs (*P* < 0.001) compared to the 5′IGRs (Fig. 2d inset). There was a modest but significant overlap between the TSA-induced acetylation and altered transcription. The increased H4K8ac within the ORFs coincided with increased expression of 66 genes (hypergeometric test, *P* = 8.5E−16) and decreased expression of 91 genes (hypergeometric test, 3.5E−27) (Fig. 2e; Additional file 7: Table S3). The upregulated gene set is functionally enriched for several pathways such as *chromatin modifying proteins* and *merozoite surface proteins* and genes with maximum expression in gametocytes and merozoite stages. The downregulated gene set upon H4K8ac hyperacetylation included factors of *DNA replication*, *excision repair* and *ribosome structure*. This effect is opposite to the H4 hypoacetylation where genes of the proliferation pathways were upregulated, and merozoite surface proteins were downregulated (Fig. 1d). This further supports our model in which H4K8ac plays a central, regulatory role within the H4ac4 moiety.

### H4K8ac regulates gene expression within both euchromatin and heterochromatin

**Fig. 3:**
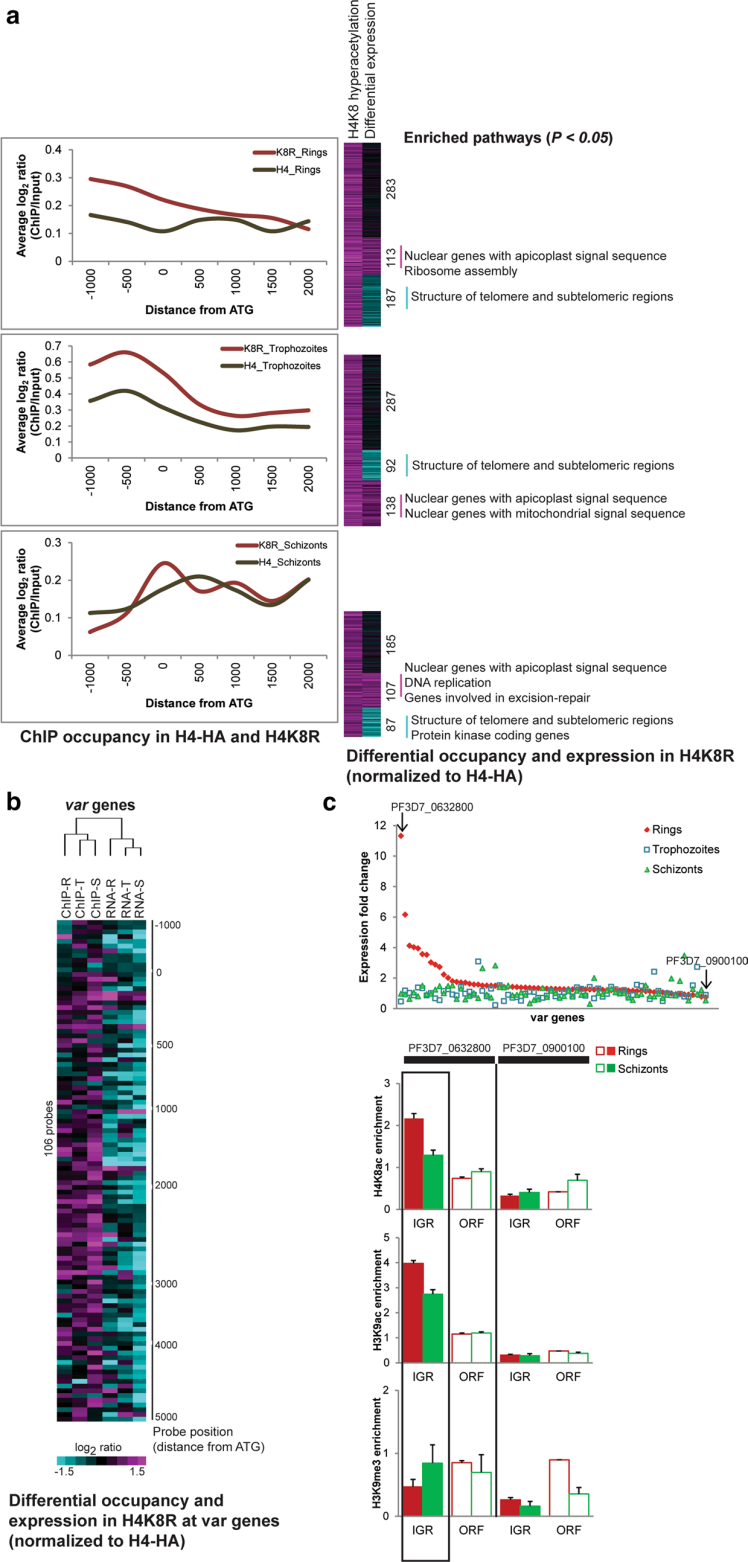
Euchromatin- and heterochromatin-linked H4K8ac. **a** ChIP-on-chip for H4-HA and H4K8R transfectants using anti-HA antibody. All microarrays were done in triplicates, and differential binding was assessed by Wilcoxon rank sum test (*P* < 0.05). *Graphs* on the *left* show average ChIP/Input log_2_ ratio of all the probes binned according to their distance from the start codon. *Heat maps* on *right* depict the probes with increased binding and change in expression in the corresponding genes in H4K8R transfectants (compared to H4-HA). Functional pathways (MPMP) significantly enriched in the up or downregulated gene sets are shown. **b** Analysis of *var* genes linked heterochromatin in H4K8R. *Heat map* represents the differential binding at *var* gene loci and differential RNA expression of corresponding genes (H4K8R normalized to H4-HA transfectants). The distance from ATG marked on the *right* depicts the microarray probe position with respect to gene start. **c** Histone marks at active/inactive *var* gene in 3D7 clone. *Graph* on *top* represents microarray gene expression values of *var* genes in one of the 3d7 clones. The highest and least expressed *var* genes are marked with the *arrow*. The lower 3 *graphs* show the ChIP enrichment by qPCR of the most dominantly expressed *var* gene and the least expressed *var* gene (input subtracted Ct values normalized to ORF of PF3D7_1240300). Primers were designed within 1000 bp on each side of the gene start. The results of the other 3 clones are shown in Additional file 4: Figure S3

Next, we wished to explore the distribution of the H4K8R mutant protein within the *P. falciparum* chromatin and correlate these to the induced transcriptional changes to find key regions of its epigenetic activity. For this we compared ChIP-on-chip results from the mutant H4K8R and unmutated H4-HA using an anti-HA antibody (Fig. 3a). Here, we observed variable occupancy patterns across the *P. falciparum* chromosomes, which suggests of the transgenic histones, displaced the endogenous histones partially with some regions being fully protected while others tolerating the mutant histone to a higher degree. Overall, there was a significant skew of the H4K8R binding to the 5′IGRs in rings and trophozoites while in schizonts, the H4K8R and H4-HA exhibit a more similar distribution. This is in sharp contrast to the TSA-induced hyperacetylation that occurs almost exclusively at the ORF of the genes (see Fig. 2d). Moreover, we observed that the increased (hypoacetylation driving) occupancy of H4K8R coincided significantly with differential expression including both up and downregulated genes. The upregulated gene set was enriched for nuclear *encoded genes responsible for apicoplast/mitochondrial import*, *DNA replication* and *repair machinery*. On the other hand, the downregulated genes with enhanced H4K8R occupancy included genes encoding the two main subtelomeric gene families involved in antigenic variations (*var* and *rifin*). This is particularly evident to the *var* genes where the enhanced H4K8R occupancy coincides with dramatically decreased transcript levels in all three stages of the IDC (Fig. 3b). This implies that the H4K8R-driven hypoacetylation at the *var* ORFs suppresses their transcription. This is surprising given that the subtelomeric regions of the *P. falciparum* chromosomes are in the heterochromatin state associated with typical heterochromatin factors such as H3K9me3 and heterochromatin binding protein HP1 [15, 35]. Our result suggests that H4K8ac, one of the main euchromatin markers in *P. falciparum*, also contributes to *var* gene regulation.

Given that the ChIp-on-chip measurements (Fig. 3b) were carried out with a parasite culture that were not selected for a single *var* gene expression, it was impossible to discern if the detected H4K8ac effect occurs specifically at the dominant transcript or all *var* genes. To distinguish between these two possibilities, we examined several isogenic clones derived by serial dilution of an in vitro culture of the *P. falciparum* 3D7 strain that exhibit a single var gene expression. These clones reflect the mutually exclusive expression with a single dominant transcript expressed at significantly higher levels in the ring stage, compared to the rest of the family (Fig. 3c; Additional file 8: Figure S3a). Using chromatin immunoprecipitation, we show that in 3 of the 4 clones, H4K8ac along with H3K9ac, is over-enriched at the intergenic region of the dominant *var* gene in the ring stage (Fig. 3c; Additional file 8: Figure S3b). On the other hand, H3K9me3 was enriched in the intergenic region of the dominant *var* transcript during the schizont stage, while in the ring stage this modification was mostly present in the ORF (Fig. 3c; Additional file 8: Figure S3b). Taken together, these results indicate that H4K8ac contributes to transcriptional regulation not only in euchromatin but also within a heterochromatin context. Consistent with its linking role between growth and host–parasite interaction (see above), this modification participates in induction (and possibly mutual expression) of the main antigenic factor in *P. falciparum*, the *var* gene family.

### Genome-wide acetylation of H4K8-bound DNA follows transcriptional dynamics during the IDC

**Fig. 4:**
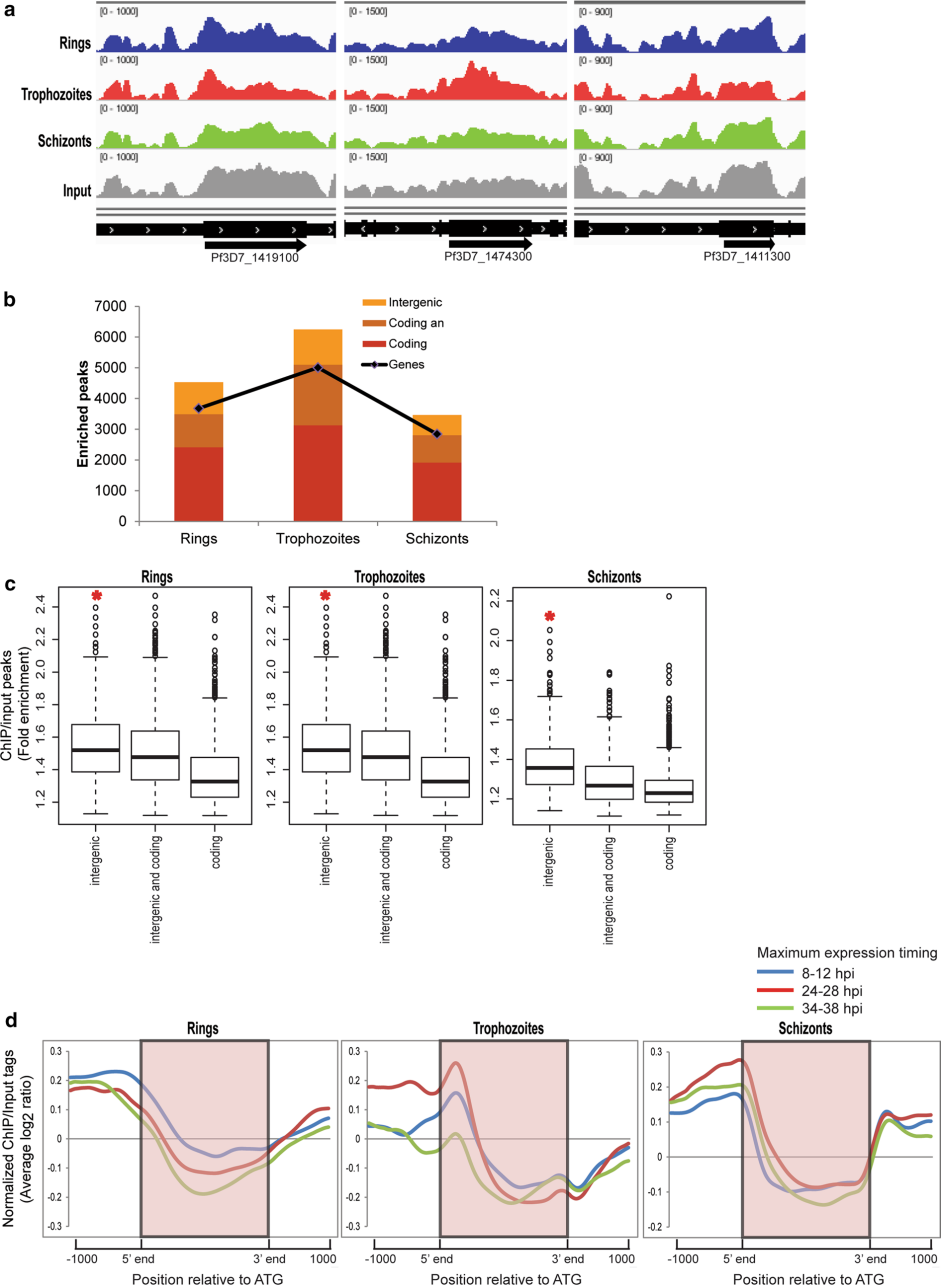
Genome-wide high-throughput ChIP-Seq to identify H4K8ac bound DNA. **a** A snapshot of the genome browser in Integrative Genomics Viewer showing normalized reads mapped to different regions of chromosome 14 representing one highly expressed gene region (*highlighted*) from each of the 3 stages. The *graphs* represent the chip over input ratios shown for gene Pf3D7_1419100, Pf3D7_1474300, Pf3D7_1411300 for rings (*blue*), trophozoites (*red*) and schizonts (*green*). Read coverage for input is shown in *gray* and the gene coordinate is marked in *black*. The tracks were normalized to library size such that the height of each track represents mapped read coverage. **b** *Bar graph* showing the number of H4K8ac peaks (identified by MACS using a *q*-value cut-off of 20%). The *line* (*graph*) represents number of genes corresponding to the peaks. **c** *Box plots* representing enrichment fold change of H4K8ac peaks covering intergenic regions, coding regions or both. The H4K8ac peaks and input peaks were normalized to the total number of reads and ratio of H4K8ac versus input peaks was calculated. *Denotes that IGR occupancy is significantly higher than ORF occupancy (Wilcoxon rank sum test, *P* < 1e−15). **d** H4K8ac binding assessed by binning the total normalized read counts (ChIP-normalized read coverage/input-normalized read coverage) into equal number of bins covering 1000-bp upstream, the gene coding body and 1000-bp downstream of each gene. The resulting profiles are plotted for genes with maximum expression at 8–12, 24–28 and 34–38 hpi corresponding to the stage when the cells were harvested for immunoprecipitation

Next, we carried out chromatin immunoprecipitation (ChIP)-coupled high-throughput sequencing (ChIP-Seq) throughout the *P. falciparum* IDC to investigate the chromosomal distribution of H4K8ac with high resolution. H4K8ac-immunoprecipitated DNA from highly synchronized parasites at the ring (8–12 hpi), trophozoite (24–28 hpi) and schizont (34–38 hpi) stages was subjected to massively parallel sequencing to obtain an average genome coverage ranging from 18–29X and 296–476X using MiSeq and HiSeq, respectively (Additional file 1). The normalized ChIP over input tag counts calculated for 2 kb region around the start codon (from 1 kb upstream to 1 kb downstream) of each gene showed correlation > 0.8 between MiSeq and HiSeq runs (Additional file 9: Figure S4a). Figure 4a shows examples of read counts spread across 3 genes highly expressed in ring, trophozoite and schizont stage, respectively. A total of 4648, 6306 and 3515 peaks were obtained for the ring, trophozoite and schizont stage HiSeq samples, respectively (Fig. 4b; Additional file 10: Table S4). In all 3 IDC developmental stages, we could observe H4K8ac occupancy peaks that were fully within the ORF or 5′IGRs, while others overlapped both. The identified H4K8ac occupancy peaks reside either within or in intergenic regions of 52, 92 and 68% genes in the ring, trophozoite and schizont stages, respectively (Fig. 4b). These results suggest that H4K8ac is predominant in the trophozoite stage where its occupancy peaks associate with the largest number of genes. There is a higher overall sequence coverage generated by the ChIP-Seq reads in the 5′IGRs followed by the peaks overlapping both 5′IGRs and ORFs and that followed by peaks exclusive to the ORFs (Fig. 4c). This is in good agreement with the previous ChIP-on-chip results showing that the majority of H4K8ac is associated with 5′IGRs with lesser occupancy within ORF [21]. We confirm this by ChIP-on-chip measurements with the identical same samples used for the RNA-Seq and showed that H4K8ac exhibits the highest occupancy within 5′IGRs with the highest signal being detected in the trophozoite stage (Additional file 9: Figure S4b).

To evaluate this further, we analyzed the H4K8ac ChIP-Seq results in the context of transcriptional stage-specificity for each gene (Fig. 4d). Indeed, in the first part of the IDC, the ring and trophozoite stages, H4K8ac showed maximum occupancy, predominantly at the 5′IGRs (1 kb upstream of the translational start) of transcriptionally active genes in both IDC stages, respectively. However, in schizonts, H4K8ac remains predominantly at the trophozoite-specific genes and increases at schizont genes only marginally. Strikingly, there is a significant shift in the positioning of H4K8ac along the gene structures throughout the IDC. In the ring stage, H4K8ac is mainly associated with relatively distal 5′IGRs, while in the trophozoite stage, there is a narrow peak distributions at the translational start sites, skewed slightly toward the 5′ termini of the ORFs (Fig. 4d). In the schizont stage, H4K8ac appears to “retreat” back to the more distal 5′IGRs, but a significant proportion of its occupancy still overlaps with the translational start sites. There is also a considerable rise in H4K8ac occupancy at the 3′IGRs in schizonts compared to the trophozoites (Fig. 4d, left two panels). Interestingly, in spite of the temporal shifts and the changing levels of the H4K8ac, the overall gene-based positional profiles of the H8K8ac were highly similar for all genes in each stage regardless of their transcriptional stage-specificity. Taken together, these results provide further evidence that H4K8ac is associated with transcriptional regulation of *P. falciparum* genes during the IDC, particularly regulating genes expressed during the mid-sections of the IDC, the trophozoite stage. At this stage, H4K8ac may contribute to transcriptional activation by repositioning its occupancy from 5′IGRs into the 5′termini of the ORFs.

## Discussion

It is now clear that in *P. falciparum*, gene regulation, in some way, is responsive to the dynamic pattern of histone marks that undergoes multitude of changes through the asexual life cycle [4, 20]. The unique plasticity of the distribution of histone marks during *P. falciparum* development is unseen in other eukaryotes. Here, we have demonstrated an important role for the histone H4 acetylations, especially at lysine 8 position, to be crucial in gene regulation in *P. falciparum*. It was previously shown that the activating histone marks are abundant and widely distributed in *P. falciparum* genome, but only some coincide with the transcriptional dynamics. However, H4K8ac plays a unique role in *P. falciparum* biology that is distinct from H3K9ac and the other activating histone marks. Although all of them are marks of transcriptionally active euchromatin in *P. falciparum*, correlation of H4K8ac with RNA polymerase II occupancy [38] and response of H4K8ac to HDAC inhibitors and DNA damaging agents is unique [39, 40]. The results presented here highlight the highly dynamic character of H4K8ac in the genome and provide first direct evidence about its importance for transcriptional reprogramming during the *P. falciparum* IDC. A certain threshold level of H4K8ac is likely required in both euchromatin and heterochromatin to allow the correct transcriptional regulation that employs both activating and suppressing factors. Here, we wish to argue that H4K8ac is the key dynamic component of the tetra-acetylation state of histone 4 (H4ac4) that ultimately functions as a main epigenetic effector, presumably via binding to trans-factors (“chromatin readers”). Although little is known about chromatin readers in *P. falciparum*, these may include proteins with conserved chromatin binding domains such as the bromodomain carrying proteins PfBDP1 and PfBDP2 that were recently shown to regulate transcription of genes of merozoite invasion [37]. Alternatively, these may involve other unique *Plasmodium* genes that were proposed to also function as chromatin readers despite of an apparent absence of conserved chromatin binding domains such as Pf14-3-3 that was shown to bind phosphorylated H3 [45].

Clonally variant multigene (CVM) families impart phenotypic diversity to *P. falciparum,* which is essential for its survival and immune evasion. There is ample evidence that single active *var* gene is enriched with H3K9ac and H3K4me3 [23, 25]. The association of H4ac with the active *var* gene was seen in a ChIP-assay followed by dot blot [46] but has never been confirmed in a more systematic way. In addition, PfMYST, the *P. falciparum* histone acetyl transferase, which acetylates histone H4 at K5, 8, 12 and 16, occupies active *var* promoter [47]. We demonstrate that H4K8 acetylation, apart from euchromatin, is another putative component of the heterochromatin environment, showing over-enrichment at the upstream region of the dominant *var* gene along with H3K9ac. In one of the studies, clonally variant multigene family genes showed a similar occupancy profile across the IDC to ring stage genes for all histone marks (including H3K9ac) except H4ac and H3Kme3 serving as the activation and repressive marks, respectively, at CVM genes [22]. This shows that association of H3K9ac and H4K8ac at the *var* genes or other clonally variant multigene family genes might have distinct roles. While both H3K9ac and H4K8ac are required for the mutually exclusive expression of dominant members, H4K8ac is required to overall regulate the expression of CVM genes.

The most significant observation made by our study is the inverse transcriptional relationship between the proliferation genes and genes of host–parasite interaction governed by H4ac4 and H4K8ac, specifically. The increased MR in case of H4K8R and H4ac4R mutants supports the model of H4K8ac playing the role of main regulator in this process. The enhanced MR is likely a reflection of the increased number of invasive merozoites and/or their viability, presumably boosted by the upregulation of the proliferation-linked genes. This observation tempts us to speculate that the *Plasmodium* cells have the ability to regulate their growth and (inversely) antigenic presentation both of which is controlled epigenetically. This is consistent with a previously conceived model in which the asexually growing *Plasmodium* parasite is in a rapidly proliferating cellular state [48], which upon casual triggering undergoes a cascade of events leading to developmental stage transitions. These triggers include several factors of physiologic environment to which the parasite can respond in its natural conditions within human host [1]. All these factors were previously shown to trigger epigenetic factors of gene expression presumably affecting the parasite physiology as a response. In 2 of our previous studies, artemisinin-resistant parasites were marked by overexpression of genes belonging to ribosome assembly and maturation [49, 50]. Upregulation of invasion-related rhoptry and merozoite surface proteins is seen during reduced expression of multidrug resistant genes Pfcrt and Pfmdr1 [51]. As such, the reduced expression of these genes during H4 hypoacetylation might be mimicking a putative resistant mechanism leading to better survival of the parasites. HDAC inhibitor-induced H4K8ac hyperacetylation is also mimicked when the cells are treated with artemisinin [40]. In light of these results, it will be interesting to investigate the potential role of H4K8ac in parasite’s adaptation to its natural host during both individual- and population-level infections.

Targeting chromatin remodeling and mechanisms of epigenetic regulation of transcription is believed to have a high potential for development of new malaria intervention strategies. TSA and other HDAC inhibitors have been previously evaluated as anti-malaria drugs due to their promising activity and selectivity seen in vitro and in vivo against *Plasmodium* parasites [52]. In accordance with the role of H4K8ac to be the major hallmark of regulation among the other histone H4 modifications, it was also found to be the most responsive modification to HDAC inhibitors in *P. falciparum*. The transient hyperacetylation was copied in expression as well as H4K8 binding to DNA. The return of hyperacetylation to pre-treatment conditions after drug removal supports the notion that these drugs are fast metabolized in the cell followed by decrease in their effective concentration [53], indicating the reversible effect to be a more general property of HDAC inhibitors. However, the irreversible effect on some histone acetylations tempts us to speculate that these two types of events are mediated by distinct histone modifications potentially generated by two distinct HDAC enzymes: PfHDAC1 for the reversible changes of euchromatin [44, 54] and the PfHDA2 for the irreversible changes involved in the gametocyte conversion [55]. Our hypothesis is supported by the fact that the small number of genes that remained de-repressed even after TSA removal belonged to gametocyte and merozoite stages. Our results confirm the role of histone modifications in exo-erythrocytic stages, and HDAC inhibitors have shown to cause hyperacetylation of *P. falciparum* gametocyte histone proteins [56]. Overall, our study opens avenue to explore HDAC inhibitors and other compound which target histone acetylations especially H4K8ac for developing novel drugs against multiple stages of malaria parasites.

## Conclusions

*Plasmodium falciparum* exhibits unique molecular mechanisms controlling chromatin remodeling as well as mechanisms that link epigenetic markers with transcription. Targeting of these molecular mechanisms has a high potential for development of new malaria intervention strategies. With our efforts to look at the combinatorial effects of histone marks on expression patterns, we can hope to decipher the histone code underlying complex regulatory make-up of these parasites and identify important elements of disease development. Our studies demonstrate the unique role of H4K8ac in maintaining the chromatin environment in *P. falciparum.* A threshold level of this acetylation most likely is required to keep the euchromatin or heterochromatin in a transcriptionally active state.

## Methods

### Parasite culture and drug treatments

*P. falciparum* strain 3D7 cells were cultured and synchronized under standard conditions [57]. Parasitemia was determined either by microscopic counting or cell sorting in the flow cytometer LSR Fortessa X-20 (BD Biosciences) using Hoechst 33342 (Sigma) [58].

### Drug treatments

*Plasmodium falciparum* cells were treated with IC_90_ values of apicidin (70 nM) or TSA (50 nM) at 5% parasitemia and 2% hematocrit for 6 h at 20-24 hpi. DMSO was used as a vehicle control. For wash-off experiments, drug-treated cells were pelleted down and washed with RPMI at least twice before culturing in fresh media without drug for specified time intervals and subsequently harvested for protein extraction, RNA extraction or chromatin immunoprecipitation. All experiments were done in either duplicates or triplicates.

### Plasmid construction and transfection

Histone H4 gene was mutated such that the targeted lysine (K) residues (K5, K8, K12, K16) were replaced with arginine (R). These amino acid exchanges were previously shown to preserve the positive charge but prevent acetylation [59, 60]. For this, plasmid pBCamR-3HA [35] was modified to create HA-tagged version of wild-type or mutated histone H4 gene in all constructs. Mutations in H4 gene were introduced by amplifying complete H4 gene of *P. falciparum* using forward primer carrying the respective change in nucleotide sequence (see Additional file 1). Unmodified H4 or H4 carrying mutations were cloned upstream of 3HA at *Bam* H1/*Nhe* 1 sites of pBcamR-3HA to make a fusion with the 3HA tag epitope and transfected into *P. falciparum* using the blasticidin-driven regulatable transgene expression systems [61]. *Plasmodium falciparum* strain 3D7 was transfected as described [62] to carry episomal copies of the plasmids in presence of selection marker blasticidin. Once the transfectants were stably maintained, the concentration of blasticidin was increased from 2.5 to 10 µg/ml in a dominant-negative selection system, which allowed the dominant expression of HA-tagged H4 protein.

### Protein extraction and immunodetection

For total protein, parasitized RBCs were lysed with 0.1% saponin and washed 2–3 times with PBS. Parasite pellets were resuspended in Laemmli SDS sample buffer, incubated at 100 °C for 10 min and centrifuged at maximum speed for 10 min to recover the supernatant.

Nuclear fractionation was done as described [35]. Briefly, saponin-lysed parasite pellets were incubated in cell lysis buffer CLB (20 mM HEPES (pH7.9), 10 mM KCl, 1 mM EDTA, 1 mM EGTA, 0.65% NP‐40, 1 mM DTT, protease inhibitors (Complete TM, Roche Diagnostics)) for 5 min on ice. Nuclei were pelleted at 5000 rpm, washed twice with CLB and digested with 300U MNase (Fermentas) in digestion buffer DB (20 mM Tris‐HCl, pH7.5, 15 mM NaCl, 60 mM KCl, 1 mM CaCl2, 5 mM MgCl2, 5 mM MnCl2, 300 mM sucrose, 0.4% NP‐40, 1 mM DTT, protease inhibitors) for 20 min at 37 °C. Soluble low salt nuclear fractions were recovered by centrifugation for 10 min at 13,000 rpm. Remaining nuclear debris was washed twice in DB and resuspended in high salt buffer HSB (20 mM HEPES (pH 7.9), 800 mM KCl, 1 mM EDTA, 1 mM EGTA, 1 mM DTT, protease inhibitors) by vortexing for 20 min at 4 °C. High salt nuclear fraction was recovered; after centrifugation for 5 min at 13,000 rpm, the high salt nuclear fraction was saved. The insoluble pellet was solubilized in SDS extraction buffer [2%SDS, 10 mM Tris‐HCl (pH 7.5)] by vortexing for 20 min at room temperature.

Acid extraction of histones was modified from the original protocol [63]. Briefly, the insoluble nuclear pellet containing DNA and histones was treated overnight with 0.25 M HCl at 4 °C. The acid-extractable protein was precipitated with trichloroacetic acid (TCA), washed in ice-cold acetone, and resuspended in Laemmli sample buffer.

For immunoblotting, identical amount of total protein lysates separated by 12% SDS-PAGE were transferred onto nitrocellulose membrane. Western blot analyses were carried out using primary antibodies probed against the core histone modifications (or HA from Sigma) obtained from Millipore, Upstate and horseradish peroxidase-conjugated secondary antibody from GE Healthcare. Polyclonal PfHDAC1 anti-serum [39], and actin (Millipore) were used as loading controls. Enhanced chemiluminescence kit was used for detection according to manufacturer’s instructions (Santa Cruz biotechnology, INC).

Immunofluorescence was carried out as described [64] using primary antibodies against HA (1:200) or H4K8ac (1:1000) and fluorophore-conjugated secondary antibodies (Invitrogen). Slides were visualized under Carl Zeiss LSM 510 Confocal Laser Scanning

Microscope.

### RNA preparation

Parasitized RBC pellets containing synchronized parasites from different stages were harvested and stored at −80 °C. RNA extraction, cDNA synthesis and amplification of cDNA were carried out as illustrated [65].

### Chromatin immunoprecipitation

Formaldehyde cross-linked chromatin was sonicated for 8 cycles or 20 cycles in case of ChIP-on-chip or ChIP-Seq, respectively, and immunoprecipitated as described [21].

### Microarray hybridizations and data analysis

#### Hybridization

Random amplification of immunoprecipitated DNA as well as input DNA for microarray was carried out for 30 cycles as described [65]. Equal amounts of Cy5 and Cy3 labeled ChIP and input DNA, respectively, were hybridized on *P. falciparum* custom arrays containing 5402 50-mer intergenic oligonucleotide probes and 10,416 70-mer ORF probes representing 5343 coding genes [66]. For RNA analysis, cDNA labeled with Cy5 was mixed with equal amounts of Cy3-labeled cDNA made from RNA from 3D7 parasites of all stages. The hybridizations were done on arrays containing *P. falciparum* ORF probes only. All hybridizations were performed on the Agilent hybridization system, and microarray scanning was done using Power Scanner (Tecan, Austria). Data were acquired using GenePix Prov6.0 software (Axon Instruments, USA).

#### Data analysis

The microarray data were normalized using the Limma package of R [67]. Briefly, LOWESS normalization was applied to all spots on each array followed by quantile normalization between arrays. Spots with flags >0 and median foreground intensity >1.5-fold median background intensity for either channel were included. The normalized log2 ratio of Cy5 versus Cy3 channel was used to present the fold change. The gene expression ratio was calculated by averaging the ratios for all probes mapping to the ORF of a gene.

Functional analyses were carried out to calculate functionally enriched pathways in a given dataset (using the functional gene annotation by MPMP [68]). Over-representation of pathways as compared to their respective frequency in the genome was calculated based on hypergeometric test (*P* < 0.05).

### Quantitative real-time PCR

For ChIP validation, qPCR was carried out on immunoprecipitated and input DNA samples. ChIP enrichment was calculated by using the ∆Ct method (Ct of immunoprecipitated target gene—Ct of input target gene) where Ct is the threshold cycle. All PCRs were done in duplicates or triplicates. Validation of invasion and proliferation genes was done by reverse transcribing RNA from H4-HA and H4K8R parasites (rings, trophozoites and schizonts) into cDNA. All qPCRs were performed using either Applied Biosystems SYBR Select Mix or SYBR Green PCR Master Mix (Bio-Rad) according to the manufacturer’s instructions.

### High-throughput ChIP-Seq

#### Library preparation

Purified DNA from H4K8ac immunoprecipitated chromatin of ring, trophozoite and schizont stages as well as sonicated genomic input DNA (schizont stage input DNA) was used to prepare ChIP-Seq libraries. Libraries for ChIP-Seq were prepared using NEBNext Ultra DNA library prep kit from Illumina according to manufacturer’s instructions with a few modifications in amplification. Libraries were amplified for 3 PCR cycles (15 s at 98 °C, 30 s at 55 °C, 30 s at 62 °C) followed by 7–9 PCR cycles (15 s at 98 °C, 30 s at 63 °C, 30 s at 65 °C) using KAPA HiFi HotStart Ready Mix (Kapa Biosystems, Woburn, MA). 150-bp paired end reads were obtained using HiSeq 2500 (Illumina).

#### Data processing

Sequence reads were aligned to the *P. falciparum* genome (downloaded from Gene DB, Jul2015) using BWA, version 0.7.12 [69]. After adapter trimming using skewer version, 0.1.125 [70], and removal of PCR duplicates, uniquely aligned reads (mapping quality >20, the probability of incorrect mapping <0.01) were extracted out for further study.

#### Data visualization

The processed data were visualized using integrative genomics viewer (IGV) interface [71]. ChIP-Seq profiles for gene models were generated using total reads counts. For this, total read coverage at 50-bp intervals was determined for regions spanning 1 kb upstream of start codon and 1 kb downstream of stop codon. Read coverage within ORF was calculated in 30 bins with the interval size approximate to 50 bp (the average length of genes in the whole genome is 1500 bp). Finally, for every library, the read coverage of each bin was normalized by the corresponding read coverage of that bin in the control input library.

#### Peak calling

The enrichment of histone occupancies was determined by peak calling algorithm built in MACS, version 2.1 [72] applied with parameters—nomodel-*q* 0.25—broad—slocal 500.

## Additional files

**Additional file 1.** (a) Vector map (b) ChIP-Seq coverage (c) Primer sequences used in this study (d) Supplemental figure and table legends.

**Additional file 2: Figure S1.** *P. falciparum* transgenic lines for mutations in H4 acetylations (related to Fig. 1).

**Additional file 3: Table S1.** Genes differentially expressed in the transfectants (related to Fig. 1).

**Additional file 4. Figure S2.** Differentially expressed pathways in the transfectants (related to Fig. 1).

**Additional file 5. Table S5.** Parasite age estimation from RNA expression data of the transfectants (related to Fig. 1).

**Additional file 6. Table S2.** Genes that remain deregulated after removal of TSA (related to Fig. 2).

**Additional file 7. Table S3.** Overlap between H4K8ac binding and expression changes during TSA treatment (related to Fig. 2).

**Additional file 8. Figure S3.** Histone marks at dominant var genes (related to Fig. 3).

**Additional file 9. Figure S4.** ChIP coupled to high throughput sequencing (related to Fig. 4).

**Additional file 10. Table S4.** MACS identified peaks for H4K8ac ChIP-Seq (related to Fig. 4).

## Abbreviations

ac: acetylation
me: methylation
IDC: intraerythrocytic developmental cycle
PTM: post transcriptional modification
ORF: open reading frame
IGR: intergenic region
HDAC: histone deacetylase inhibitor
TSA: trichostatin A
ChIP: chromatin immunoprecipitation
bp: base pair
