# Supplementary material for: Histone 4 lysine 8 acetylation regulates proliferation and host–pathogen interaction in Plasmodium falciparum
Source: Epigenetics Chromatin. 2017 Aug 22;10:40. doi: 10.1186/s13072-017-0147-z (PMC5568195; doi:10.1186/s13072-017-0147-z)
Supplement: Supplementary file 1 — Additional file 1. (a) Vector map (b) ChIP-Seq coverage (c) Primer sequences used in this study (d) Supplemental figure and table legends. [file 13072_2017_147_MOESM1_ESM.doc]

**a) Plasmid used to introduce mutations in histone H4 gene**

**Mutations in histone H4 gene**

Shown below are the H4 sequences with mutations highlighted for each construct. The H4 gene in all constructs is HA tagged.

***P. falciparum* Histone H4 acetylation specific lysine K (AAA, AAG) mutated to arginine  R (AGA)**

**H4-HA**: ATG TCA GGA AGA GGT AAG GGA GGT AAA GGT TTG GGA AAA GGA GGA GCT AAG

**H4K5R**: ATG TCA GGA AGA GGT AGA GGA GGT AAA GGT TTG GGA AAA GGA GGA GCT AAG

**H4K8R**: ATG TCA GGA AGA GGT AAG GGA GGT AGA GGT TTG GGA AAA GGA GGA GCT AAG

**H4K12R**:ATG TCA GGA AGA GGT AAG GGA GGT AAA GGT TTG GGA AGA GGA GGA GCT AAG

**H4K16R**:ATG TCA GGA AGA GGT AAG GGA GGT AAA GGT TTG GGA AAA GGA GGA GCT AGA

**H4ac4R**:ATG TCA GGA AGA GGT AGA GGA GGT AGA GGT TTG GGA AGA GGA GGA GCT AGA

**b) ChIP-sequencing performed in this study**

| **S.No** | **Samples** | **Mapped reads** | **Unique reads** | **Coverage (X)** |
| --- | --- | --- | --- | --- |
| 1 | MiSeq_Input | 6,606,926 | 4,830,755 | 28.75 |
| 2 | MiSeq_Rings | 5,638,758 | 4,077,404 | 24.88 |
| 3 | MiSeq_Trophozoites | 4,446,739 | 3,058,177 | 17.91 |
| 4 | MiSeq_Schizonts | 5,455,877 | 4,007,751 | 24.53 |
| 5 | HiSeq_Input | 88,288,178 | 80,357,117 | 476.71 |
| 6 | HiSeq_Rings | 85,660,412 | 67,136,799 | 406.91 |
| 7 | HiSeq_Trophozoites | 96,469,922 | 50,422,888 | 296.18 |
| 8 | HiSeq_Schizonts | 93,262,176 | 63,179,487 | 383.86 |

c) Oligonucleotide sequences used in this study

| **Primer** | **Purpose** | **Orientation to gene** | **Sequence** |
| --- | --- | --- | --- |
| H4_F | amplify histone H4 for cloning | Forward | GCC GGATCC ATG TCA GGA AGA GGT AAG GGA GGT AAA GGT TTG GGA AAA GGA GGA GCT AAG |
| K5R_F | amplify histone H4 with mutation at lysine 5 for cloning | Forward | GCC GGATCC ATG TCA GGA AGA GGT AGA GGA GGT AAA GGT TTG GGA AAA GGA GGA GCT AAG |
| K8R_F | amplify histone H4 with mutation at lysine 8 for cloning | Forward | GCC GGATCC ATG TCA GGA AGA GGT AAG GGA GGT AGA GGT TTG GGA AAA GGA GGA GCT AAG |
| K12R_F | amplify histone H4 with mutation at lysine 12 for cloning | Forward | GCC GGATCC ATG TCA GGA AGA GGT AAG GGA GGT AAA GGT TTG GGA AGA GGA GGA GCT AAG |
| K16R_F | amplify histone H4 with mutation at lysine 16 for cloning | Forward | GCC GGATCC ATG TCA GGA AGA GGT AAG GGA GGT AAA GGT TTG GGA AAA GGA GGA GCT AGA |
| acR_F | amplify histone H4 with mutation at lysines 5,8,12 and 16 for cloning | Forward | GCC GGATCC ATG TCA GGA AGA GGT AGA GGA GGT AGA GGT TTG GGA AGA GGA GGA GCT AGA |
| H4_NOSTOP_R | amplify histone H4 for cloning | Reverse | GCC CCATGG ACCTCCAAAACCATATAAAGTTCTTCCTTGTC |
| BSD_F | RT | Forward | ACAGCGTCGCCAGCGCAGCTCTCTCTA |
| BSD_R | RT | Reverse | ATCGCGACGATACAAGTCAGGTTGCCAGCT |
| ARGINYL-TRNA SYNTHETASE_F | RT | Forward | AAGAGATGCATGTTGGTCATTT |
| ARGINYL-TRNA SYNTHETASE_R | RT | Reverse | GAGTACCCCAATCACCTACA |
| PF3D7_0632800_F_IGR | RT | Forward | ACGTTCTTGTTCTTGTTCTCG |
| PF3D7_0632800_R_IGR | RT | Reverse | AGAGGCAAATATCTTCTTCCA |
| PF3D7_0632800_F_ORF | RT | Forward | AAGAACGGGAAGAAGACGCT |
| PF3D7_0632800_ORF | RT | Reverse | AACATTTGCCACACATCGGC |
| PF3D7_0900100_F_IGR | RT | Forward | TTTCGATACGTTCTTGTTCTCG |
| PF3D7_0900100_R_IGR | RT | Reverse | GGCAAATATCTTCTTCTGTTTCTCTC |
| PF3D7_0900100_F_ORF | RT | Forward | TGAACACAGCAAATGGTCGT |
| PF3D7_0900100_R_ORF | RT | Reverse | GCACGGATTCCTTTCATTGT |
| PF3D7_1300300_F_IGR | RT | Forward | TTCAACAATAGTACGAGCAAAACA |
| PF3D7_1300300_R_IGR | RT | Reverse | TCCATACATTCATCACCTACAACA |
| PF3D7_1300300_F_ORF | RT | Forward | AACATGTAGAGCCCCAAACG |
| PF3D7_1300300_R_ORF | RT | Reverse | AATCATCTGCACAACGTGGA |
| PF3D7_0712900_F_IGR | RT | Forward | TGAATTAAACATTATACAGATCGAAAA |
| PF3D7_0712900_R_IGR | RT | Reverse | TCCCATTATTAGTCCAACAAAACA |
| PF3D7_0712900_F_ORF | RT | Forward | CGCATAAGTTGTTGGCAGAA |
| PF3D7_0712900_R_ORF | RT | Reverse | GTCATGGCTTCCCAGATTGT |
| PF3D7_0600100_F_IGR | RT | Forward | TTCAACAATAGTACGAGCAAAACA |
| PF3D7_0600100_R_IGR | RT | Reverse | CGATACCAAAGGTTTGCCATTA |
| PF3D7_0600100_F_ORF | RT | Forward | AAAGGGTGCAGAAAAACGTG |
| PF3D7_0600100_R_ORF | RT | Reverse | ACATTCTGCTTCGCTTTCGT |
| PF3D7_0712800_F_IGR | RT | Forward | TTCGATACCCTTTTGTTCTCG |
| PF3D7_0712800_R_IGR | RT | Reverse | GGCAAATATGTTCTTCCGTTTC |
| PF3D7_0712800_F_ORF | RT | Forward | CAAGGAAATCCATGCCAACT |
| PF3D7_0712800_R_ORF | RT | Reverse | TGCTCCTACCCTGTCGTCTT |
| PF3D7_0600200_F_IGR | RT | Forward | TTCAACAAATACATTCTTATTCTAC |
| PF3D7_0600200_R_IGR | RT | Reverse | TATGTCATGCATACGTGGTT |
| PF3D7_0600200_F_ORF | RT | Forward | TGCCATGCTGCACATAGTGA |
| PF3D7_0600200_R_ORF | RT | Reverse | TTGACACTCTTCCTTGGCCG |
| PF3D7_0809100_F_IGR | RT | Forward | GCATTCATAAAATCCATGAAATACA |
| PF3D7_0809100_R_IGR | RT | Reverse | CAAATATGTTCTTCCGTTTCTCTC |
| PF3D7_0809100_F_ORF | RT | Forward | GGGGGTAGGGAGTATCCTTG |
| PF3D7_0809100_R_ORF | RT | Reverse | CGCTCGTGGTCAACTCATTA |
| PF3D7_1240300_F_IGR | RT | Forward | CAAGCCACTAGTGCGAAGGA |
| PF3D7_1240300_R_ORF | RT | Reverse | GATGGCATGGATCCGGTGAT |
| PF3D7_0501600_F | RT | Forward | TGCACACACTGCAAATGTTC |
| PF3D7_0501600_R | RT | Reverse | ACTGCTCTAGCAAAACATGTATGA |
| PF3D7_0501500_F | RT | Forward | ACGTGCACATAAACCCGATA |
| PF3D7_0501500_R | RT | Reverse | AGCTCTTGCAAAACAAGCATGA |
| PF3D7_1035600_F | RT | Forward | AGCATGCTGTTACGGTTGTT |
| PF3D7_1035600_R | RT | Reverse | TGTCCAGGAGAAAAATGGCGA |
| PF3D7_0719700_F | RT | Forward | CGCAAAGATACCACGTCACC |
| PF3D7_0719700_R | RT | Reverse | TCCTCTTCCCATTGGTTGCC |
| PF3D7_0522500_F | RT | Forward | CGACTGAAGCAAAAGCAAAGGA |
| PF3D7_0522500_R | RT | Reverse | GCCATTCTTGATGCGTCTCC |

**d)** **Supplemental figure and table legends**

**Figure S1. *P. falciparum* transgenic lines for mutations in H4 acetylations (related to Figure 1). (A) Increase in plasmid copy number.** Change in plasmid copy number was determined by carrying out quantitative real time PCR (qPCR) on blasticidin S deaminase (BSD) gene and calculating the Ct value difference between cells grown at 2.5 and 10 µg/ml BSD. The Ct values were normalized to single copy arginyl-tRNA synthetase gene. **(B) Nuclear localization of HA-tagged H4 proteins**. Upper panel: Western blot was done using acid extracted histones using antiHA antibody to confirm the presence of HA-tagged H4 proteins in histone fraction. Anti-H4 antibody was used as positive control. Lower panel: Immunofluorescence detection of HA-tagged proteins in the nucleus using primary antibodies against HA and fluorophore conjugated secondary antibodies. Anti-H4K8ac was used as a positive control for nuclear localization. Cells were grown in 10 µg/ml blasticidin for both the experiments. **(C) Correlation between the control transfectants.** Microarray expression data of RNA extracted from 5 time points across the IDC for HA and H4-HA transfectants was heirarchically clustered along with the RNA extracted from 3 time points across the IDC for H4-HA (3 time points, 3 bioloical replicates used for normalizing the expression data from the transfectants with mutations). **(D)** **Correlation between the transfectants with mutations.** Heat map shows the Pearson’s correlation coefficient between the differential expression (normalized to H4-HA, average of triplicates) of all genes in the transfectants. **(E)** **Comparison between expression profiling at 2.5 and 10 µg/ml blasticidin.** RNA extraction and expresison profiling was done for the transfectants H4-HA and H4K8R grown at 2.5 and 10 µg/ml blasticidin at schizont stage. Heat map shows that transcriptional changes between H4K8R and H4-HA (both grown at blasticidin 10 µg/ml) are similar to those induced in H4K8R grown in 10 and 2.5 µg/ml of blasiticidin. On the other hand, H4-HA grown in 10 and 2.5 µg/ml of blasticidin shows very few transcriptional changes. The pathways enriched in H4K8R when grown in 10 versus 2.5 µg/ml of blasiticidin are shown on the right. (**F) Transcription of ribosomal protein coding genes.** Heat map depicts the diferential expression (H4-HA subtracted data, average of triplicates) of ribosomal protein coding genes in the transfectants. As shown in Figure 1D, the cytoplasmic ribosomal protein coding genes are mainly annotated as *genes coding for ribosome struture* , *genes coding for components involved in ribosome assembly* and *maturation and export of 60S and 40S ribosomal subunits*. The organellar ribosomal protein coding genes are mainly annotated as *ribosome structure genes*, *nulear genes with apicoplast signal sequence* and *nuclear genes with mitochondrial signal sequence* (for details see MPM pathway annotation [1]). **(G) Timing of transgene expression.** Westen blot analyses with the transgenic cell line of unmutated H4 carried out in three developmental stages ring (R), trophozoite (T) and schizonts (S). **(H)** **Comparison of transgene versus wildtype protein expression.** The protein intensities from western blot used in Figure 1A were measured using Image J. After backfround subtarction, intensities of HA-tagged proteins were comapred with endogenous proteins in the transfectants grown at both 2.5and 10 µg/ml of blasticidin. **(I) Quantitative real time PCR validation of deregulated genes in H4K8R.** Candidate genes belonging to merozoite invasion or ribosomal protein subunits down or up-regulated in H4K8R microarray data were validated using qRT PCR. Fold change expression in H4K8R comapred to H4-HA was calculated in respective genes from trophozoite and schizont stages. The graph repersents data from two individual biological replicates. RAP2: PF3D7_0501600; RAP3: PF3D7_0501500; MSP H101: PF3D7_1035600; 40S S10: PF3D7_0719700; 50SL17: PF3D7_0522500.

**Figure S2. Differentially expressed pathways in the transfectants (related to Figure 1).** Boxplots were created to represent the difference in expression for all the genes (as annotated by MPMP) belonging to the differentially expressed pathways (as shown in Figure 1D). For transfectants with mutation and empty vector control, microarray log_2_ ratio of every gene in the pathway was normalized to H4-HA carrying no mutation.

**Figure S3. Histone marks at dominant *var* genes (related to Figure 3). (A) Expression of dominant *var* gene in 3D7 clones.** RNA was extracted from 4 clones of 3D7 at ring, trophozoite and schizont stages. cDNA from the respective RNA samples was hybridized against 3D7 reference pool. The graphs represent fold change in expression of *var* genes at the 3 stages. The dominantly expressed *var* gene in each of the clone is marked with the arrow. **(B)** **H4K8ac is enriched at the intergenic regions of dominant *var* gene.** The top graph shows the expression of one dominant *var* gene and one silent *var* gene in the 4 clones of 3D7. The bottom 3 graphs show the ChIP enrichment of the same genes by qPCR (input subtracted Ct values normalized to ORF of PF3D7_1240300).

**Figure S4. ChIP coupled to high throughput sequencing (related to Figure 4). (A) Reads obtained from ChIP-Seq using H4K8ac antibody** Read counts were binned into 50 bp bins covering 1000 bp on each side of ATG for every gene. The graph shows the correlation between raw reads obtained from MiSeq and HiSeq runs for all 5420 genes. Regression coefficient (R^2^) was calculated at each of the 3 stages. **(B)** **ChIP-on-chip using H4K8ac antibody.** H4K8ac immunoprecipitated chromatin used for ChIP-Seq was also used for microarray for comparison. ChIP-on-chip profile (averaged ChIP/input log_2_ ratios) for all genes is plotted against probe position from -1000 bp to +2000 bp with respect to ATG.

**Table S1. Genes differentially expressed in the transfectants (related to Figure 1). Microarray expression analyses of transfectants across 3 stages of the IDC.** Differential expression was assessed by paired student’s T-test (*P < 0.05*). Data is represented as average log_2_ ratio of biological triplicates and normalized to the unmutated H4-HA transfectant.

**Table S2. Genes that remain deregulated after removal of TSA (related to Figure 2).** The table lists the genes that remain up or down regulated even after TSA is being washed off. The stage of the IDC when the gene is maximally expressed under normal conditions is shown.

**Table S3. Overlap between H4K8ac binding and expression changes during TSA treatment (related to figure 2).** The table lists the genes (corresponding to microarray probes on the chip), which show differential binding and are also differentially expressed between TSA treated and non-treated cells.

**Table S4. MACS identified peaks for H4K8ac ChIP-Seq (related to figure 4).**

**Table S5. Parasite age estimation (related to Figure 1).** The gene expression data of each transfectant sample was compared to a reference IDC transcriptome using Pearson’s correlation coefficient in order to map the age of the parasites post invasion [2].

**Supplemental References:**

1. Ginsburg H: **Progress in in silico functional genomics: the malaria Metabolic Pathways database.** *Trends Parasitol* 2006, **22:**238-240.

2. Mok S, Imwong M, Mackinnon MJ, Sim J, Ramadoss R, Yi P, Mayxay M, Chotivanich K, Liong KY, Russell B, et al: **Artemisinin resistance in Plasmodium falciparum is associated with an altered temporal pattern of transcription.** *BMC Genomics* 2011, **12:**391.
