# Supplementary material for: Histone 4 lysine 8 acetylation regulates proliferation and host–pathogen interaction in Plasmodium falciparum
Source: Epigenetics Chromatin. 2017 Aug 22;10:40. doi: 10.1186/s13072-017-0147-z (PMC5568195; doi:10.1186/s13072-017-0147-z)
Supplement: Supplementary file 2 — Additional file 2: Figure S1. P. falciparum transgenic lines for mutations in H4 acetylations (related to Fig. 1). [file 13072_2017_147_MOESM2_ESM.pdf]

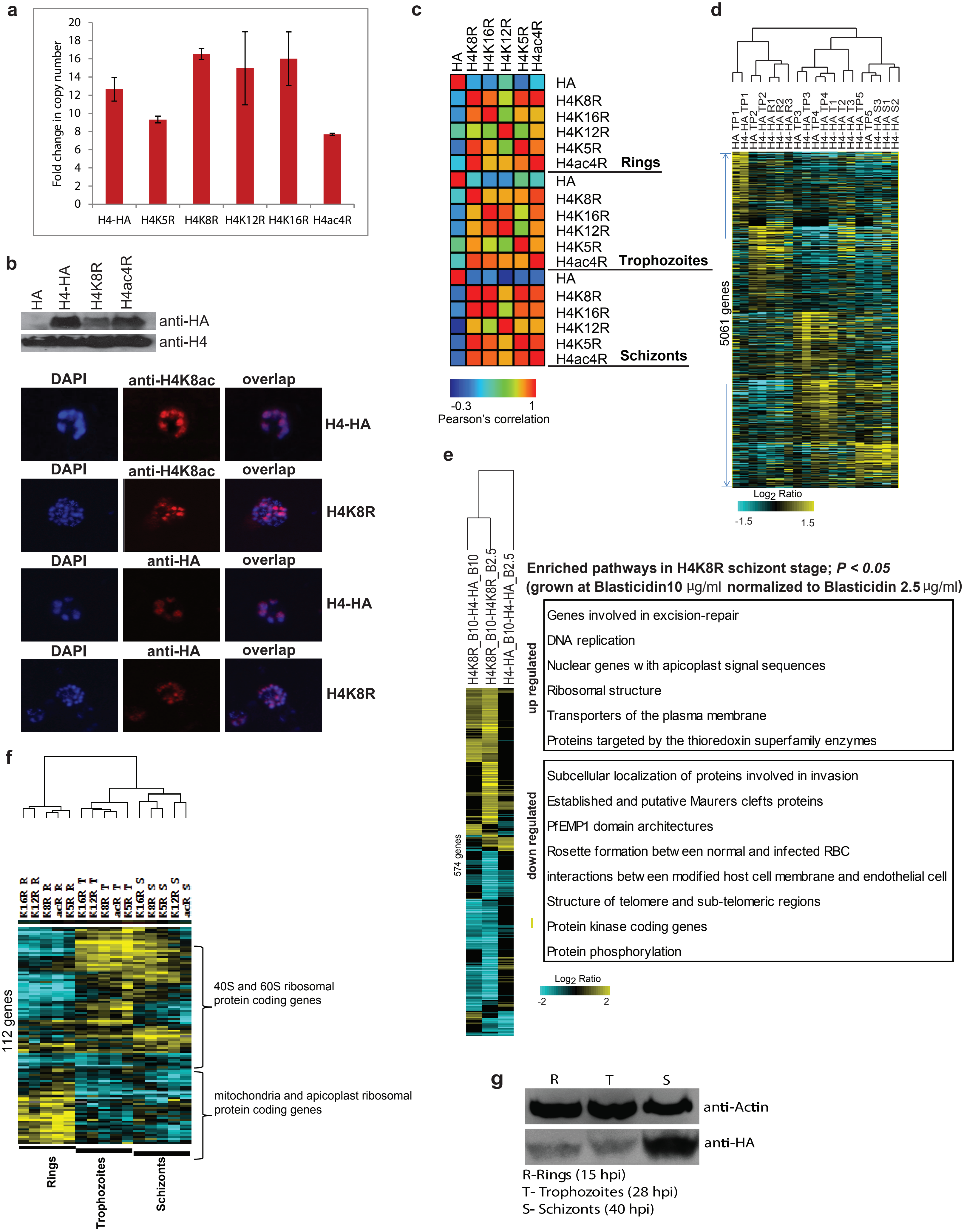

a
Fold change in copy number
20
18
16
14
12
10
8
6
4
2
0
H4-HA
H4K5R
H4K8R
H4K12R
H4K16R
H4ac4R
b
HA
H4-HA
H4K8R
H4ac4R
anti-HA
anti-H4
DAPI
anti-H4K8ac
overlap
H4-HA
H4K8R
anti-HA
c
HA
H4K8R
H4K16R
H4K12R
H4K5R
H4ac4R
Rings
Trophozoites
Schizonts
-0.3
1
Pearson's correlation
d
HA TP1
H4-HA TP1
HA TP2
H4-HA TP2
H4-HA R1
H4-HA R2
H4-HA R3
HA TP3
H4-HA TP3
HA TP4
H4-HA TP4
H4-HA T1
H4-HA T2
H4-HA T3
HA TP5
H4-HA TP5
H4-HA S3
H4-HA S1
H4-HA S2
5061 genes
Log2 Ratio
-1.5
1.5
e
H4K8R_B10-H4-HA_B10
H4K8R_B10-H4K8R_B2.5
H4-HA_B10-H4-HA_B2.5
574 genes
Enriched pathways in H4K8R schizont stage; P < 0.05
(grown at Blasticidin10 µg/ml normalized to Blasticidin 2.5 µg/ml)
up regulated
Genes involved in excision-repair
DNA replication
Nuclear genes with apicoplast signal sequences
Ribosomal structure
Transporters of the plasma membrane
Proteins targeted by the thioredoxin superfamily enzymes
down regulated
Subcellular localization of proteins involved in invasion
Established and putative Maurers clefts proteins
PfEMP1 domain architectures
Rosette formation between normal and infected RBC
interactions between modified host cell membrane and endothelial cell
Structure of telomere and sub-telomeric regions
Protein kinase coding genes
Protein phosphorylation
Log2 Ratio
-2
2
f
K16R R
K12R R
K8R R
acR R
K5R R
K16R T
K12R T
K8R T
acR T
K5R T
K16R S
K8R S
K5R S
K12R S
acR S
112 genes
40S and 60S ribosomal protein coding genes
mitochondria and apicoplast ribosomal protein coding genes
Rings
Trophozoites
Schizonts
g
R
T
S
anti-Actin
anti-HA
R-Rings (15 hpi)
T- Trophozoites (28 hpi)
S- Schizonts (40 hpi)

h

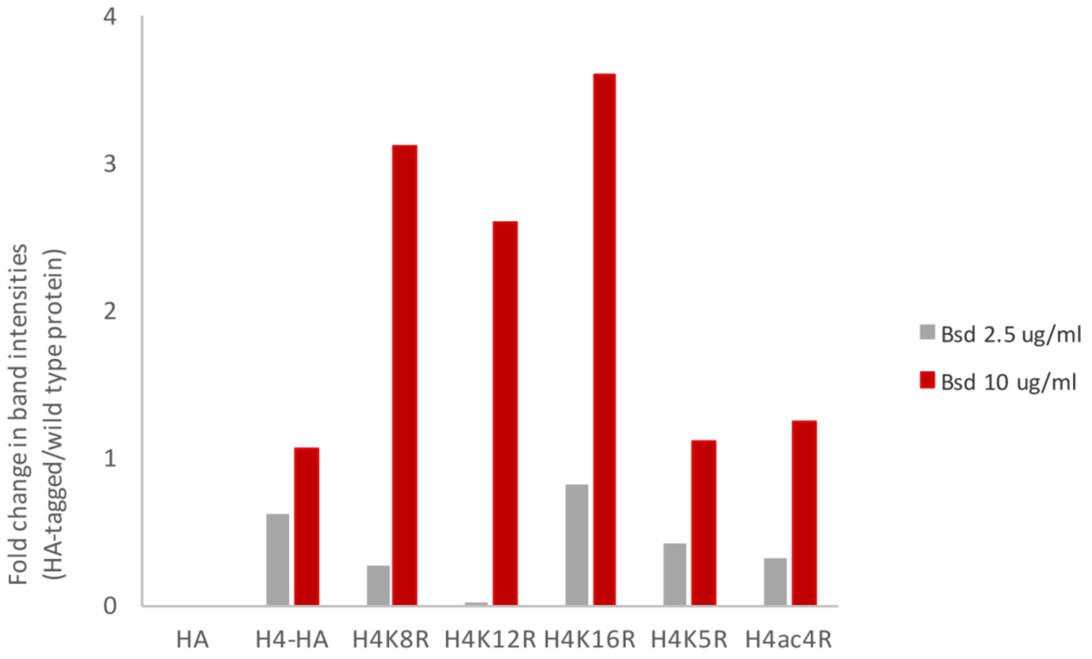

Fold change in band intensities
(HA-tagged/wild type protein)
4
3
2
1
0
HA
H4-HA
H4K8R
H4K12R
H4K16R
H4K5R
H4ac4R
Bsd 2.5 ug/ml
Bsd 10 ug/ml

i

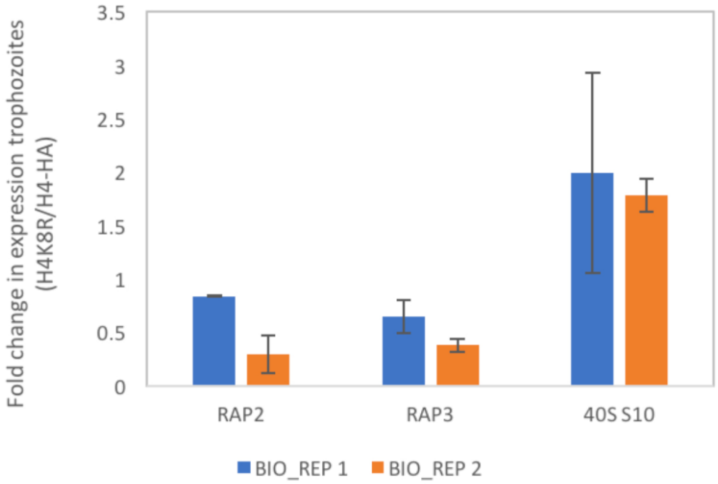

Fold change in expression trophozoites (H4K8R/H4-HA)
3.5
3
2.5
2
1.5
1
0.5
0
RAP2
RAP3
40S S10
BIO_REP 1
BIO_REP 2

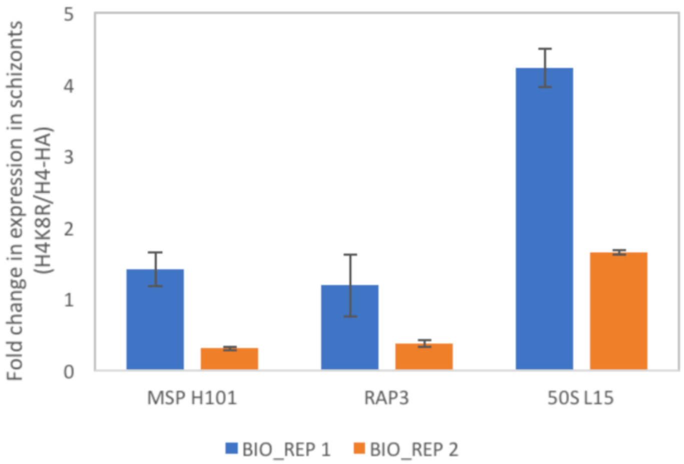

Fold change in expression in schizonts (H4K8R/H4-HA)
5
4
3
2
1
0
MSP H101
RAP3
50S L15
BIO_REP 1
BIO_REP 2
