# Supplementary material for: Histone 4 lysine 8 acetylation regulates proliferation and host–pathogen interaction in Plasmodium falciparum
Source: Epigenetics Chromatin. 2017 Aug 22;10:40. doi: 10.1186/s13072-017-0147-z (PMC5568195; doi:10.1186/s13072-017-0147-z)
Supplement: Supplementary file 4 — Additional file 4. Figure S2. Differentially expressed pathways in the transfectants (related to Fig. 1). [file 13072_2017_147_MOESM4_ESM.pdf]

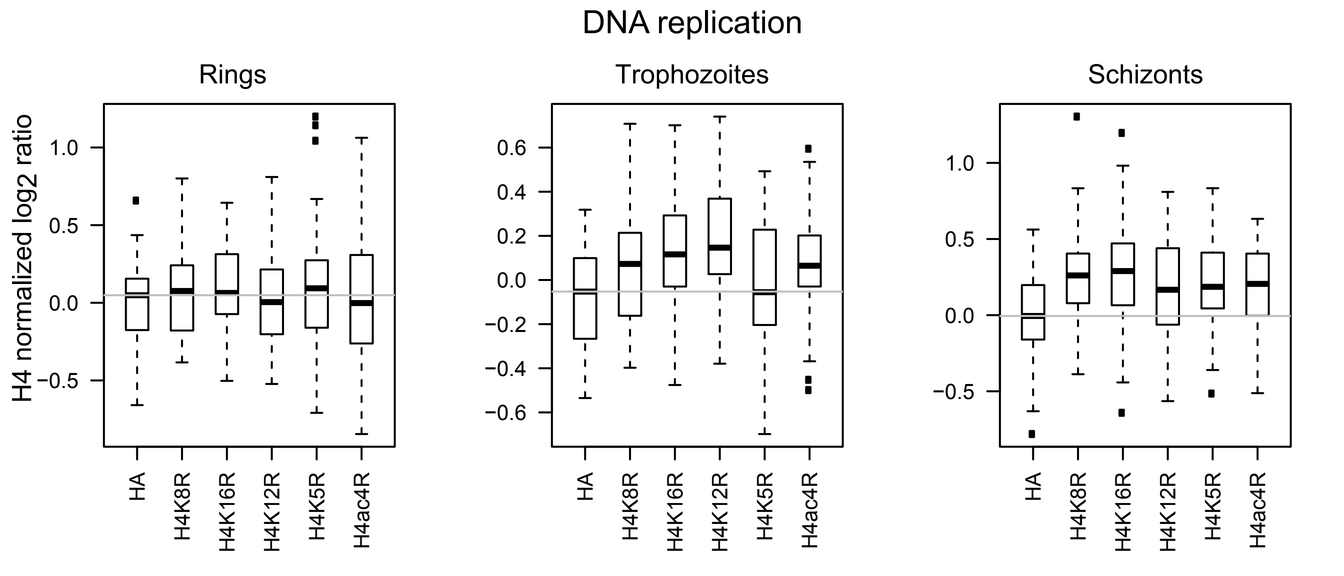
DNA replication
Rings
Trophozoites
Schizonts
H4 normalized log2 ratio
1.0
0.5
0.0
−0.5
0.6
0.4
0.2
0.0
−0.2
−0.4
−0.6
HA
H4K8R
H4K16R
H4K12R
H4K5R
H4ac4R

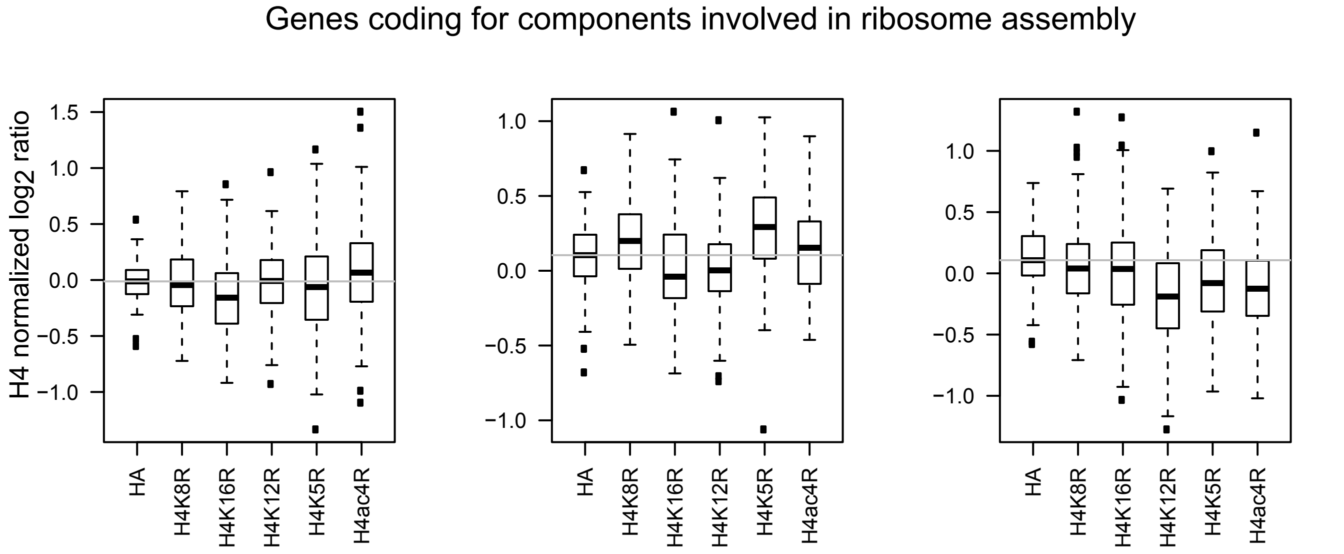

Genes coding for components involved in ribosome assembly
H4 normalized log2 ratio
1.5
1.0
0.5
0.0
−0.5
−1.0
HA
H4K8R
H4K16R
H4K12R
H4K5R
H4ac4R

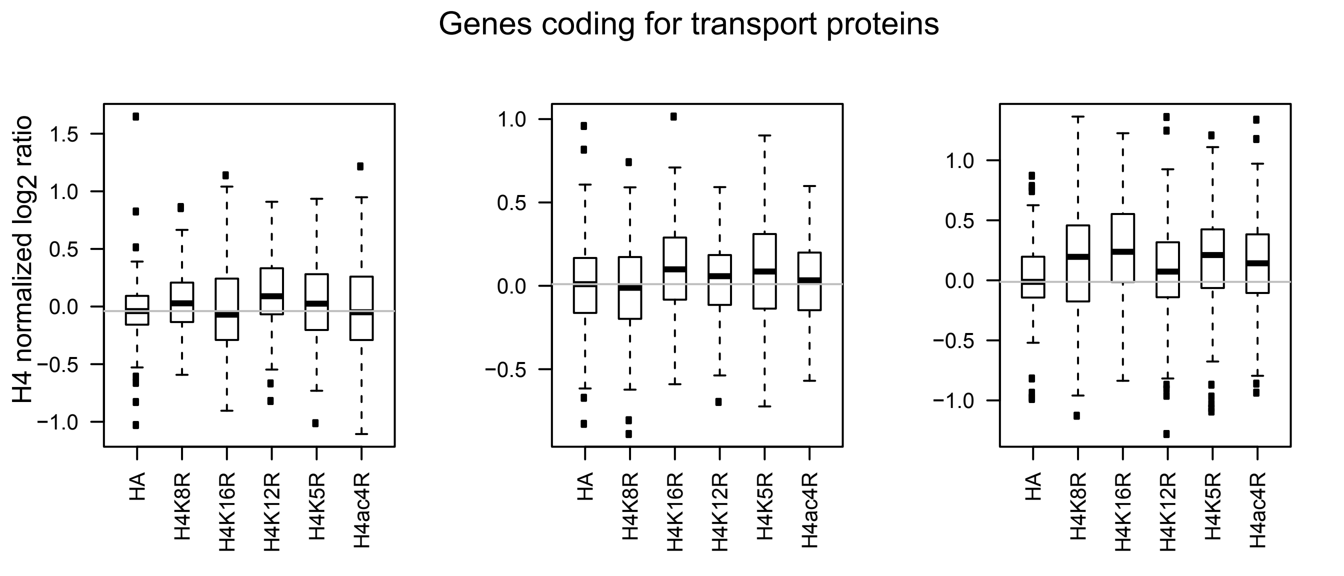

Genes coding for transport proteins
H4 normalized log2 ratio
1.5
1.0
0.5
0.0
−0.5
−1.0
HA
H4K8R
H4K16R
H4K12R
H4K5R
H4ac4R

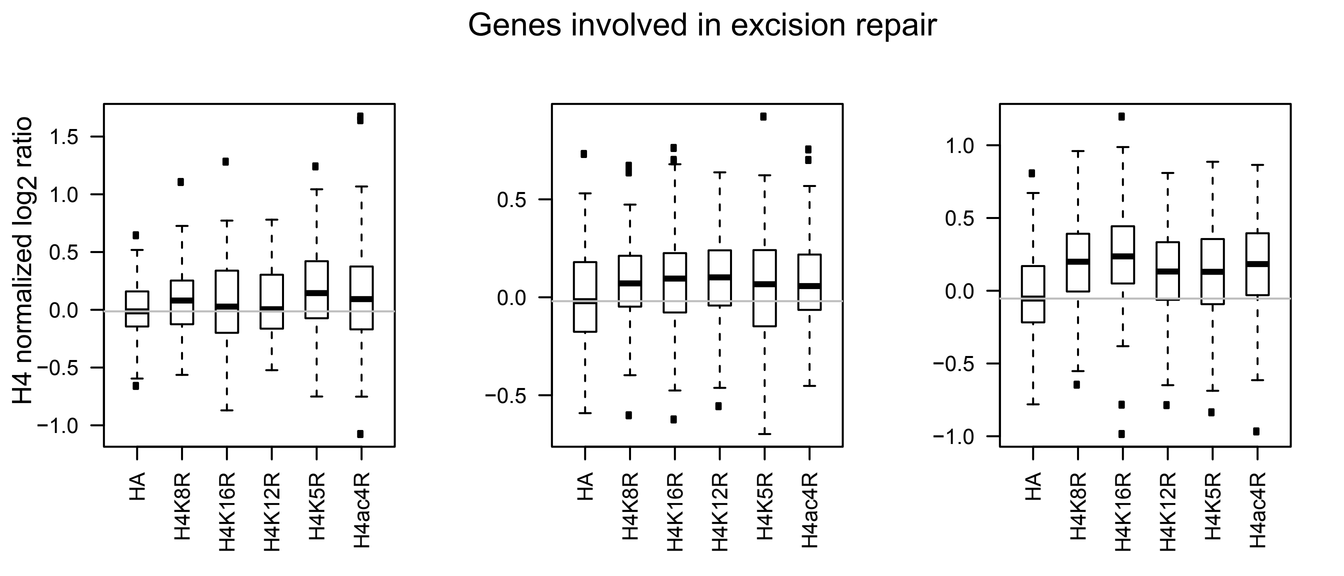

Genes involved in excision repair
H4 normalized log2 ratio
1.5
1.0
0.5
0.0
−0.5
−1.0
HA
H4K8R
H4K16R
H4K12R
H4K5R
H4ac4R

Glycolysis

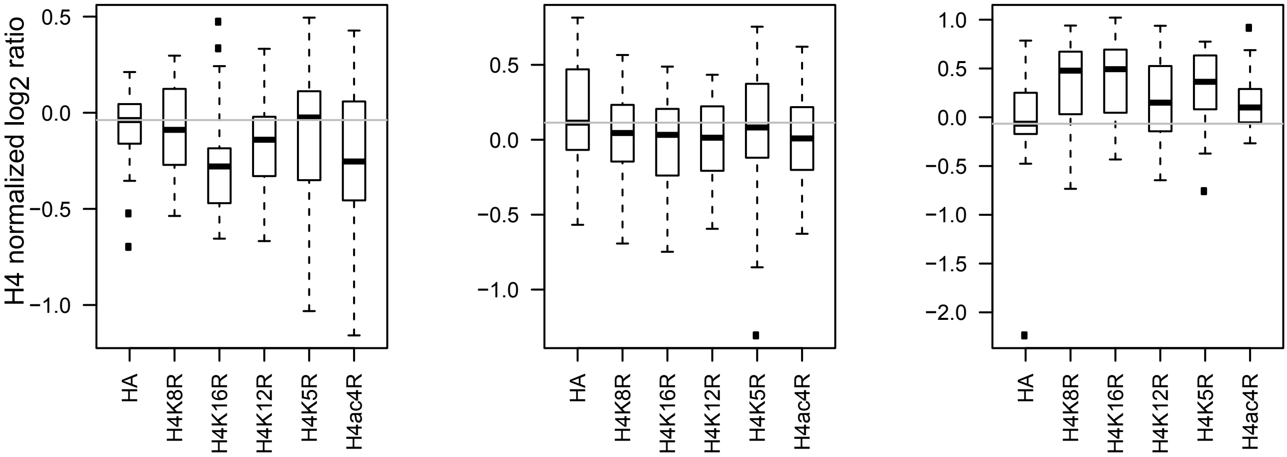
H4 normalized log2 ratio
0.5
0.0
−0.5
−1.0
HA
H4K8R
H4K16R
H4K12R
H4K5R
H4ac4R
1.0
−1.5
−2.0

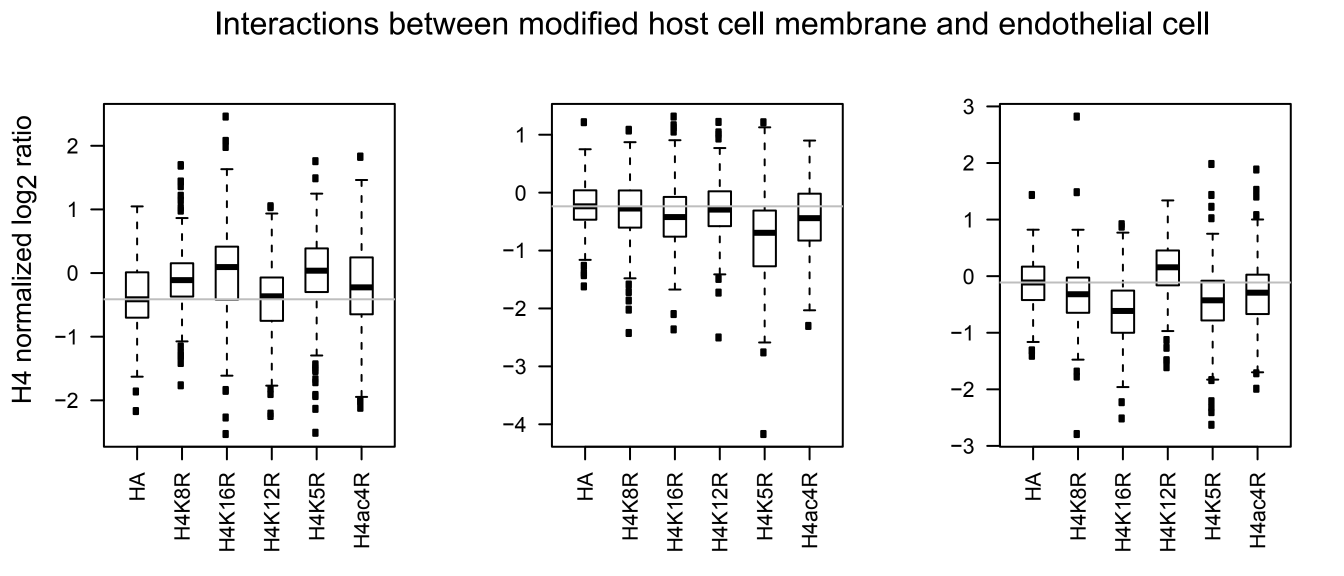

Interactions between modified host cell membrane and endothelial cell
H4 normalized log2 ratio
2
1
0
−1
−2
0
−1
−2
−3
−4
3
−3
HA
H4K8R
H4K16R
H4K12R
H4K5R
H4ac4R

Maturation and export of 60S and 40S ribosomal subunits

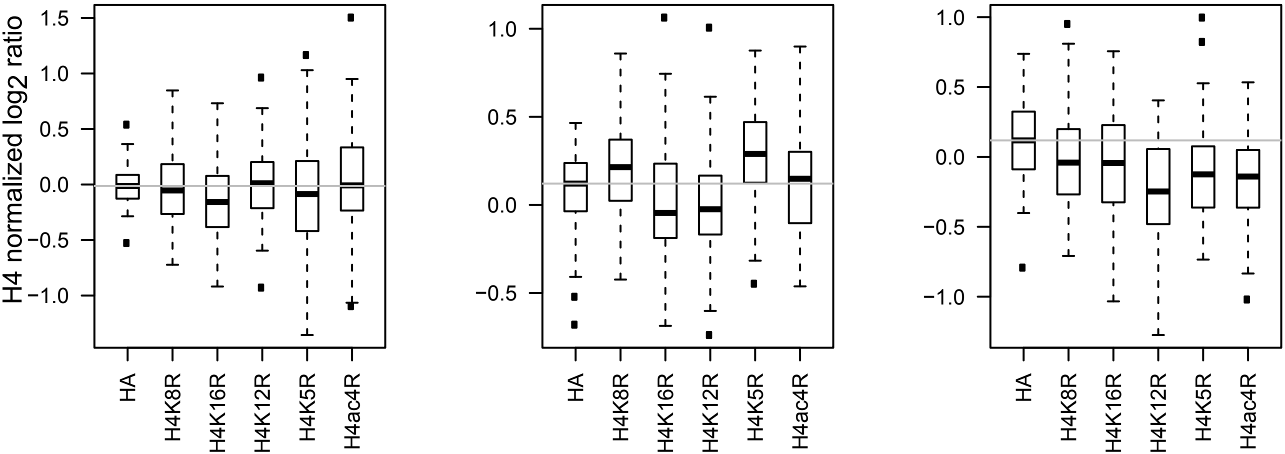

Nuclear genes with apicoplast signal sequence

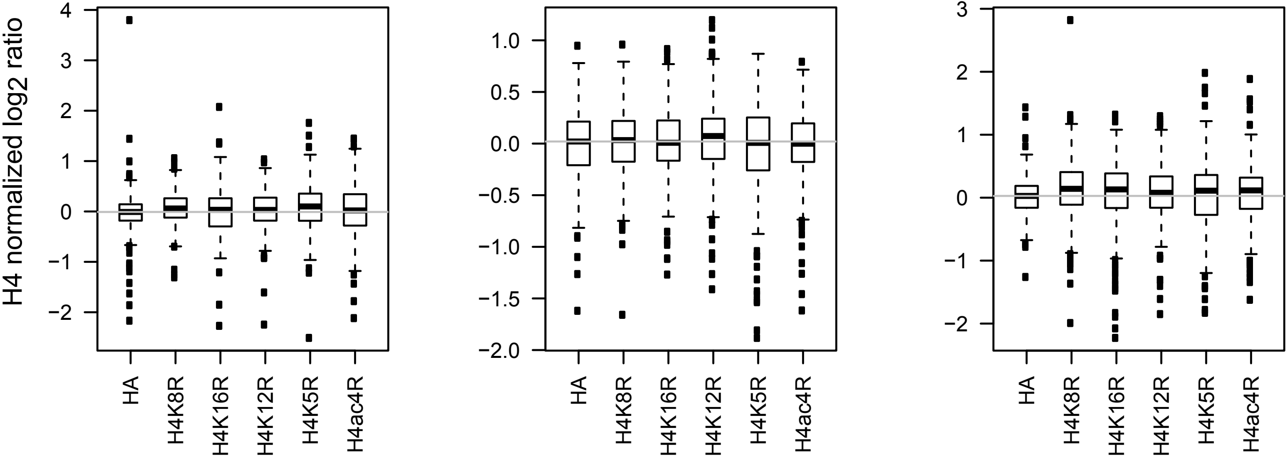

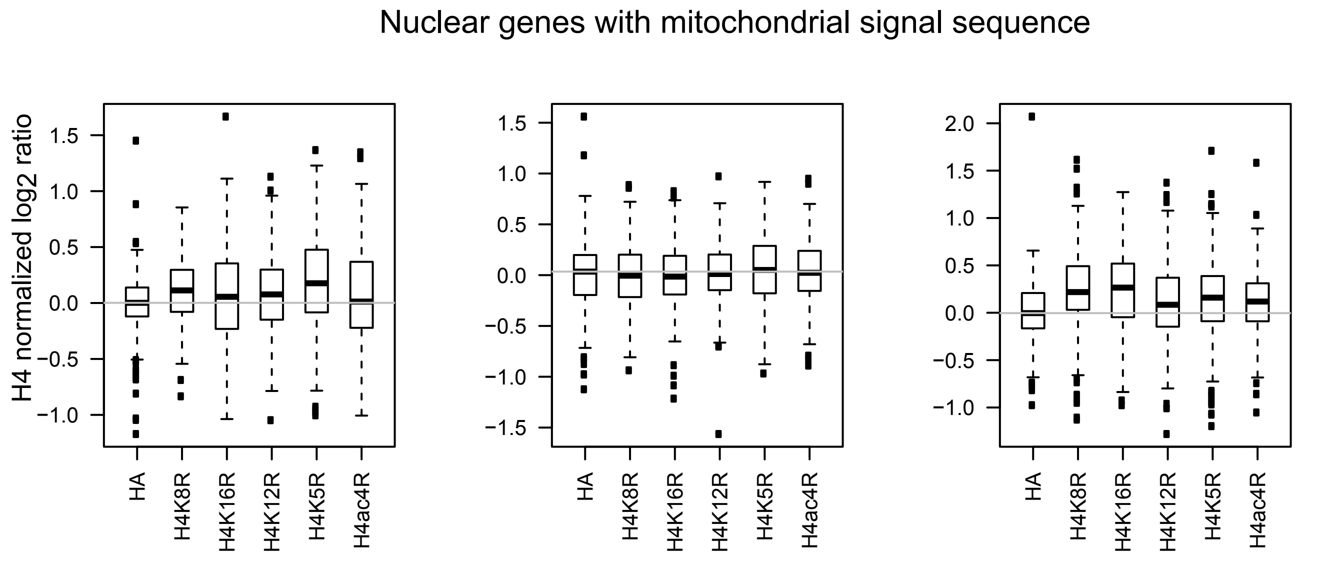
Nuclear genes with mitochondrial signal sequence
H4 normalized log2 ratio
1.5
1.0
0.5
0.0
−0.5
−1.0
−1.5
2.0
HA
H4K8R
H4K16R
H4K12R
H4K5R
H4ac4R

Peptidases and proteases

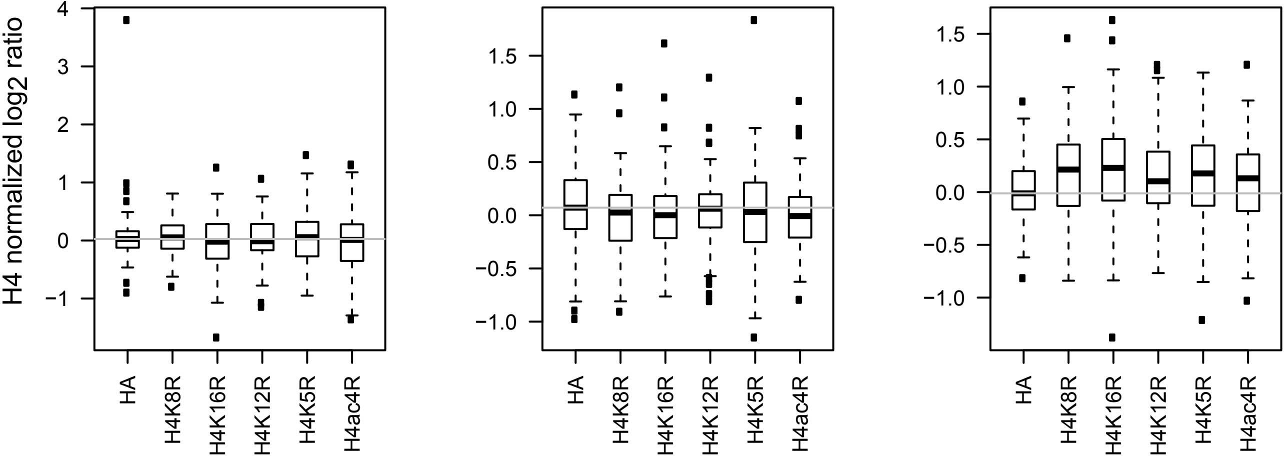

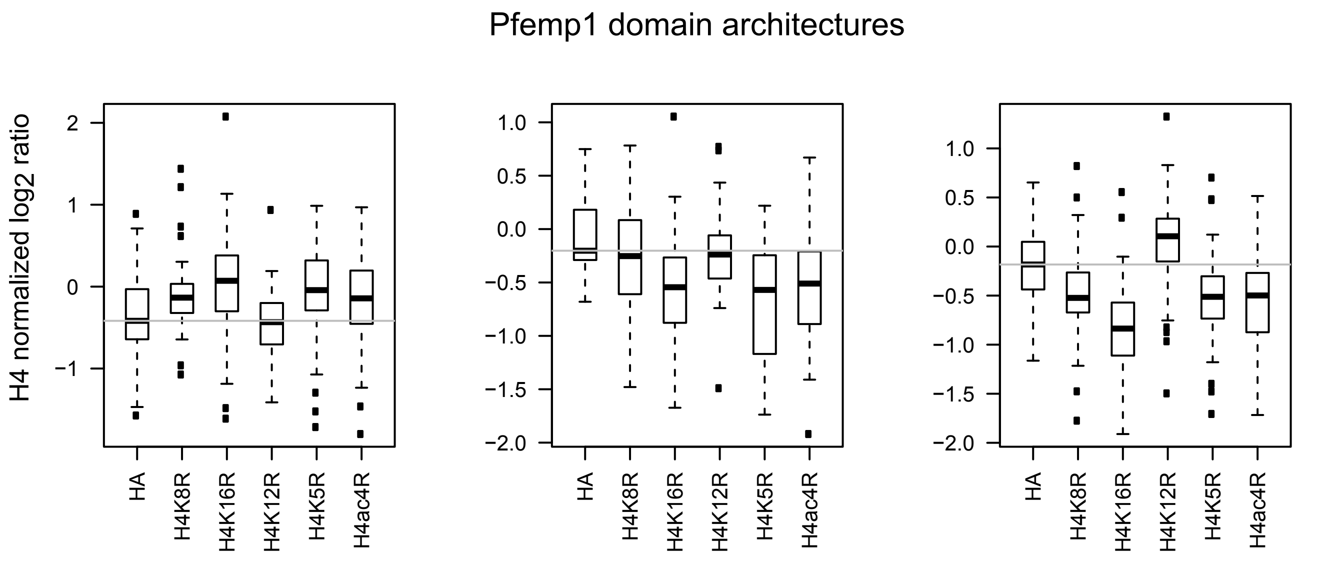

Pfemp1 domain architectures
H4 normalized log2 ratio
2
1
0
−1
1.0
0.5
0.0
−0.5
−1.0
−1.5
−2.0
HA
H4K8R
H4K16R
H4K12R
H4K5R
H4ac4R

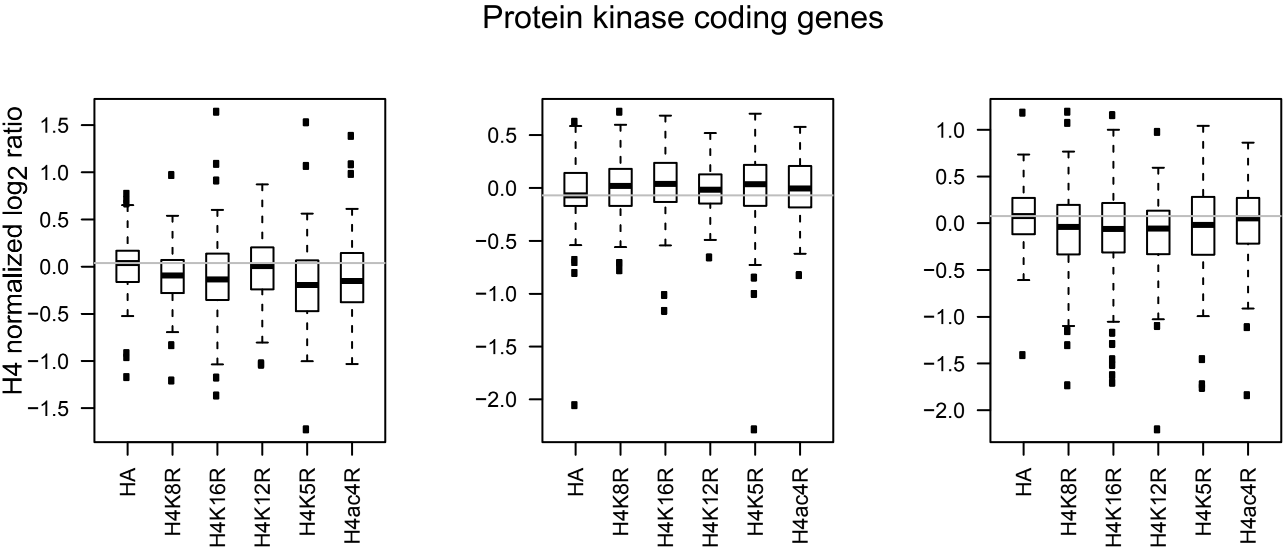
Protein kinase coding genes
H4 normalized log2 ratio
1.5
1.0
0.5
0.0
−0.5
−1.0
−1.5
−2.0
HA
H4K8R
H4K16R
H4K12R
H4K5R
H4ac4R

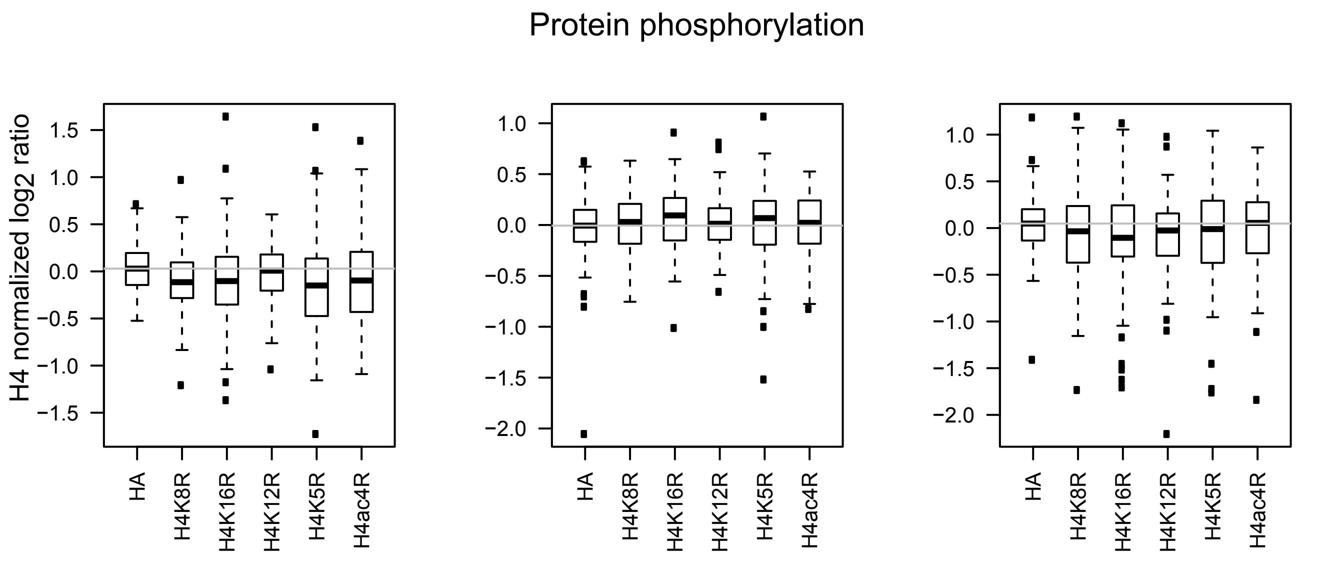

Protein phosphorylation
H4 normalized log2 ratio
1.5
1.0
0.5
0.0
−0.5
−1.0
−1.5
−2.0
HA
H4K8R
H4K16R
H4K12R
H4K5R
H4ac4R

Proteins targeted by the thioredoxin superfamily

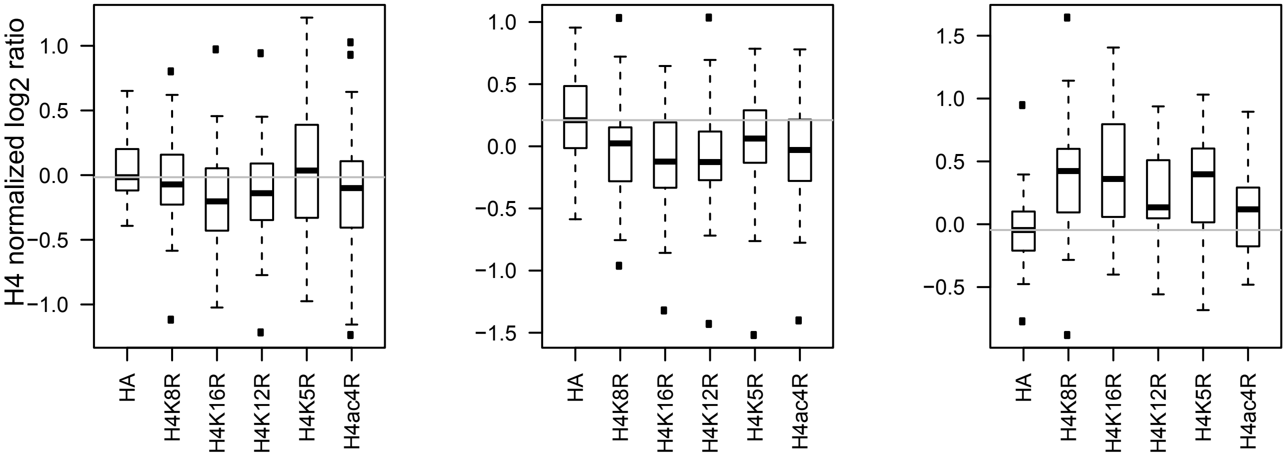

Ribosome structure

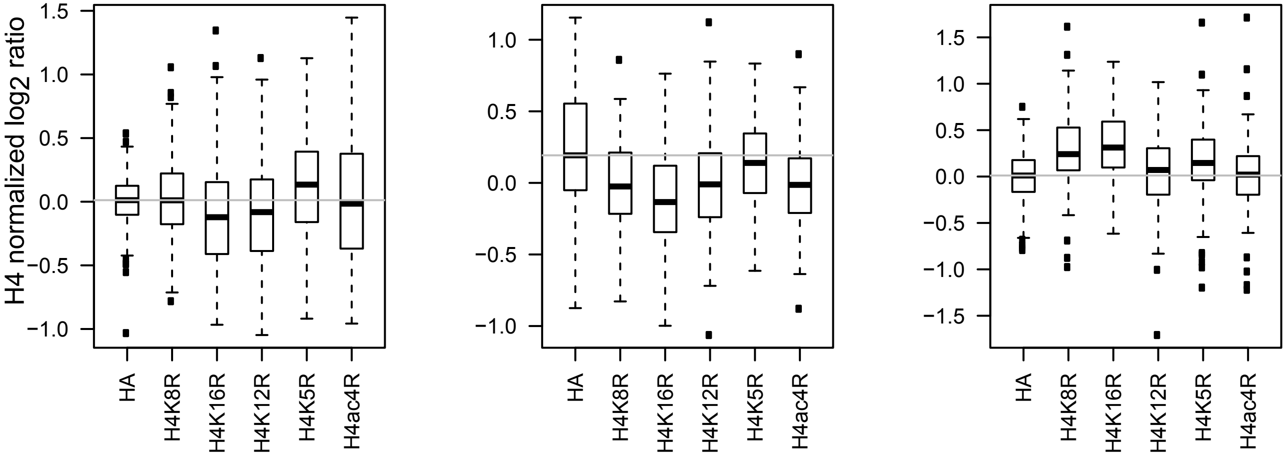

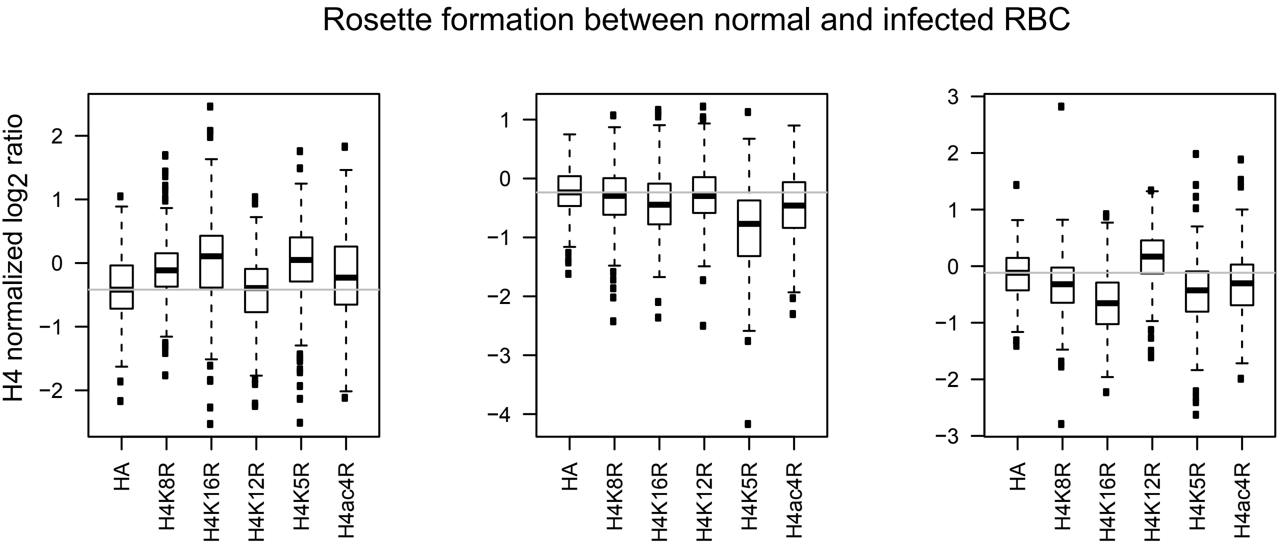
Rosette formation between normal and infected RBC
H4 normalized log2 ratio
HA
H4K8R
H4K16R
H4K12R
H4K5R
H4ac4R

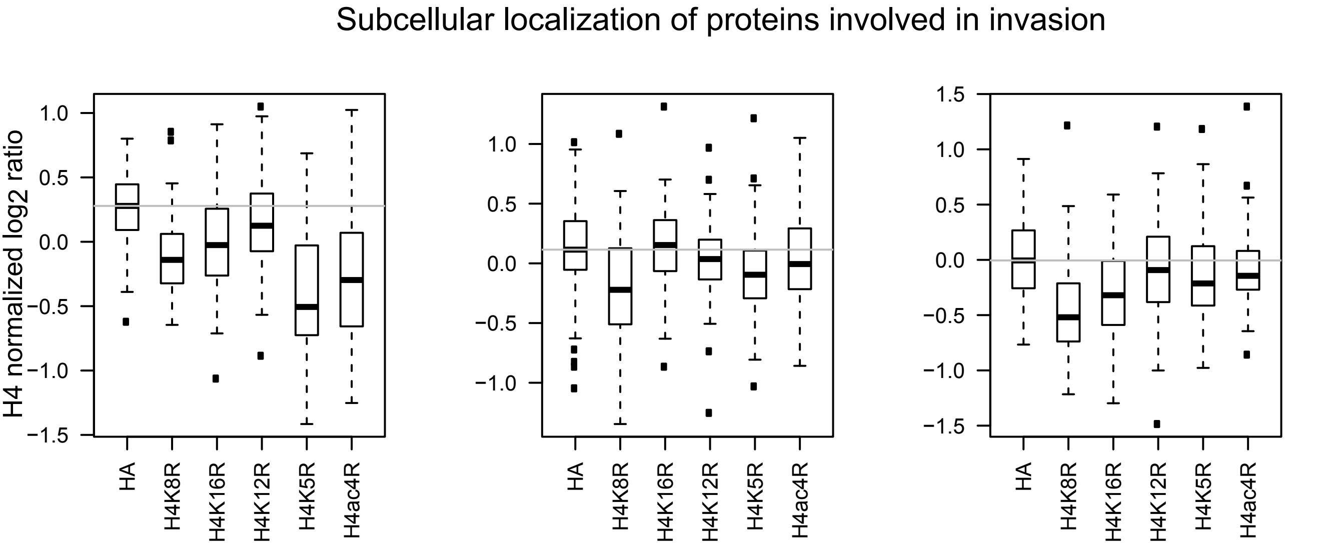

Subcellular localization of proteins involved in invasion
H4 normalized log2 ratio
1.0
0.5
0.0
−0.5
−1.0
−1.5
HA
H4K8R
H4K16R
H4K12R
H4K5R
H4ac4R
1.0
0.5
0.0
−0.5
−1.0
HA
H4K8R
H4K16R
H4K12R
H4K5R
H4ac4R
1.5
1.0
0.5
0.0
−0.5
−1.0
−1.5
HA
H4K8R
H4K16R
H4K12R
H4K5R
H4ac4R

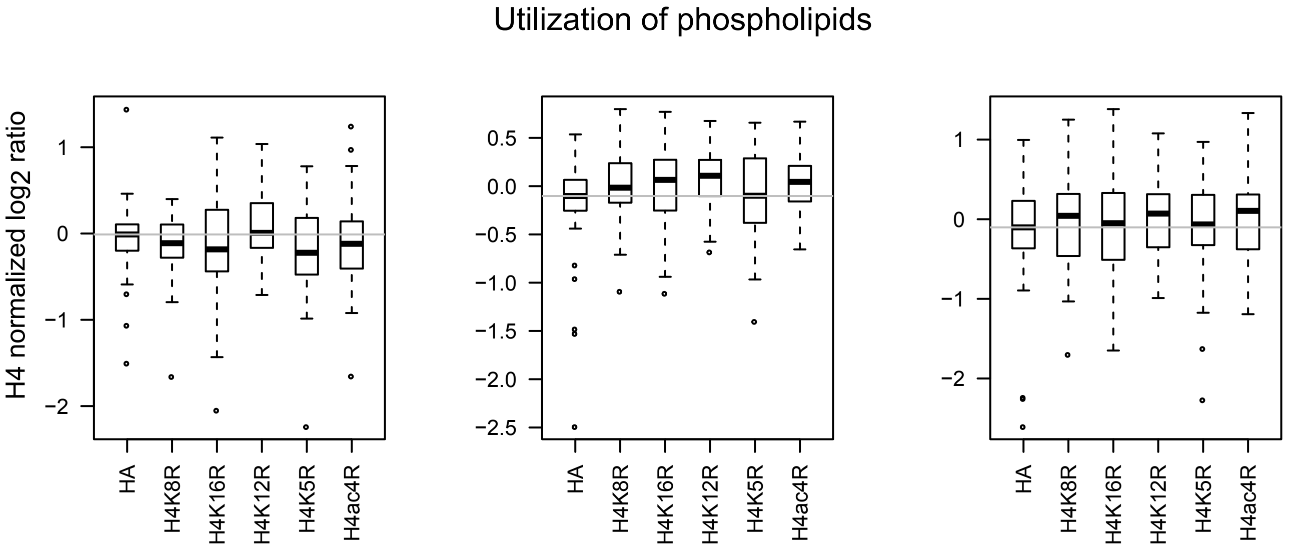

Utilization of phospholipids
H4 normalized log2 ratio
1
0
−1
−2
0.5
0.0
−0.5
−1.0
−1.5
−2.0
−2.5
HA
H4K8R
H4K16R
H4K12R
H4K5R
H4ac4R
