# Supplementary figures and images for: Histone 4 lysine 8 acetylation regulates proliferation and host–pathogen interaction in Plasmodium falciparum
Source: Epigenetics Chromatin. 2017 Aug 22;10:40. doi: 10.1186/s13072-017-0147-z (PMC5568195; doi:10.1186/s13072-017-0147-z)

a

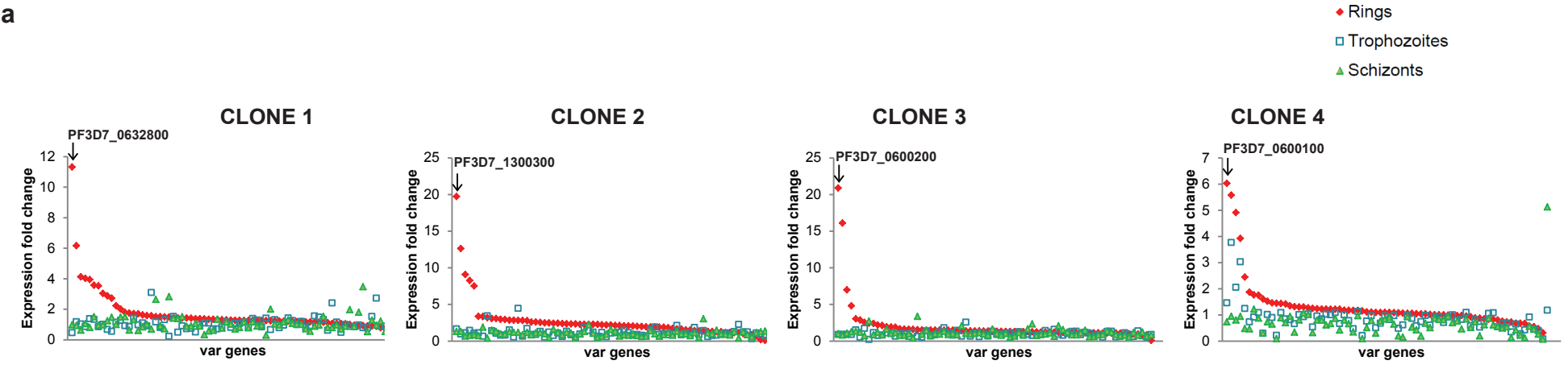

b

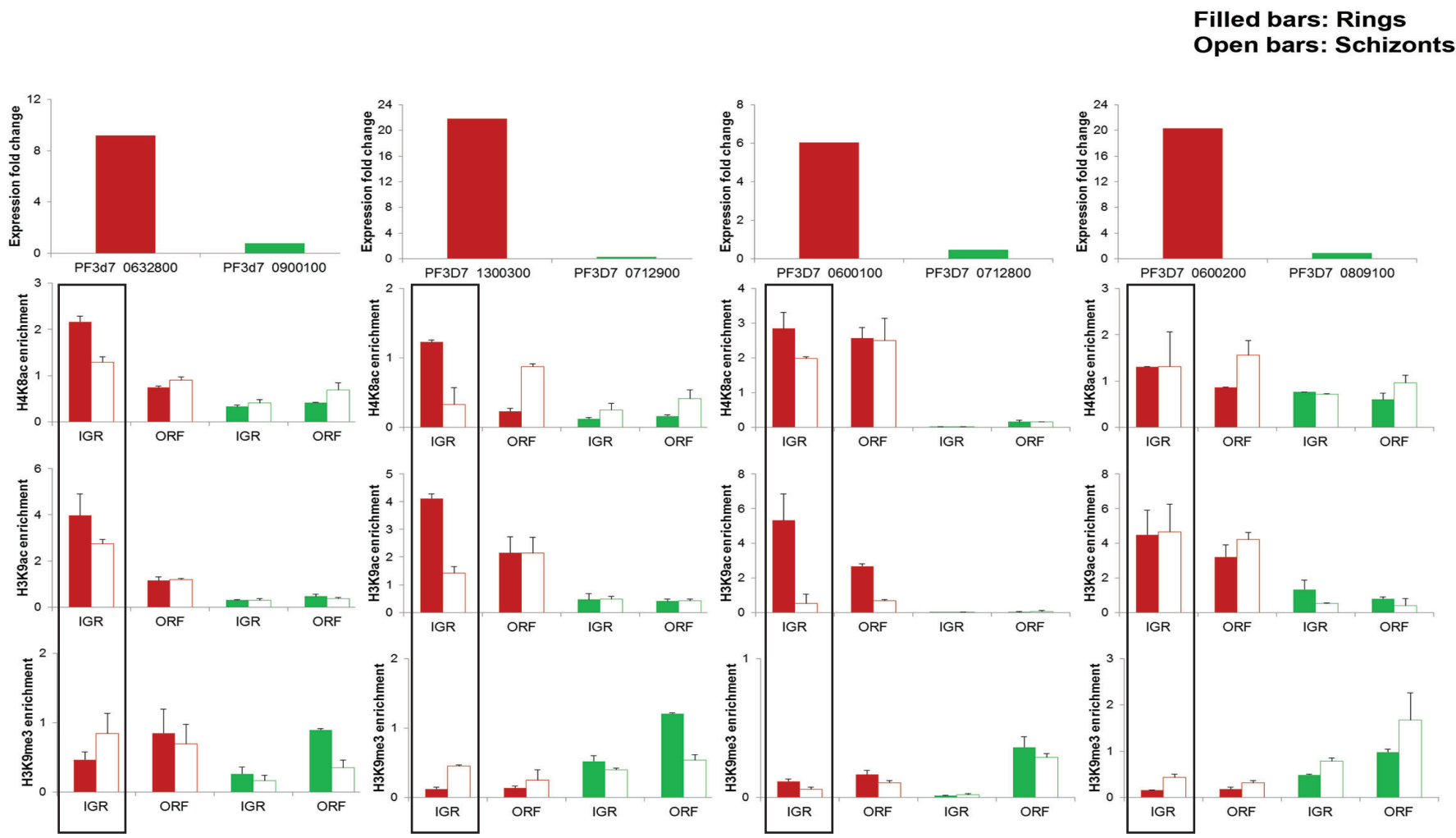

Supplement: Supplementary file 8 — Additional file 8. Figure S3. Histone marks at dominant var genes (related to Fig. 3). [file 13072_2017_147_MOESM8_ESM.pdf]

a

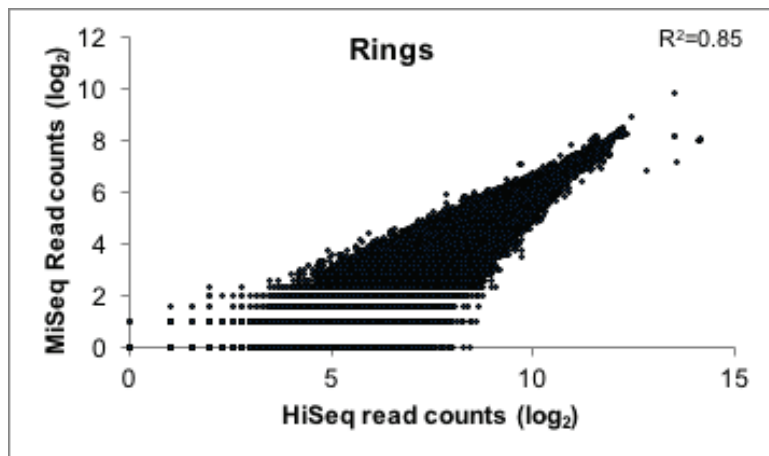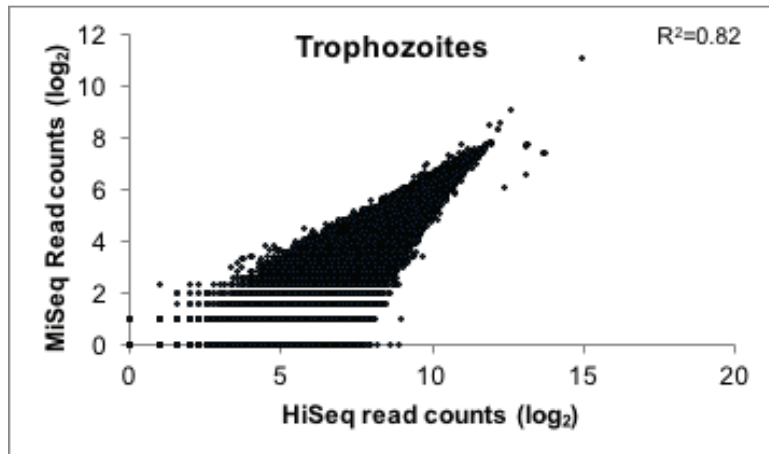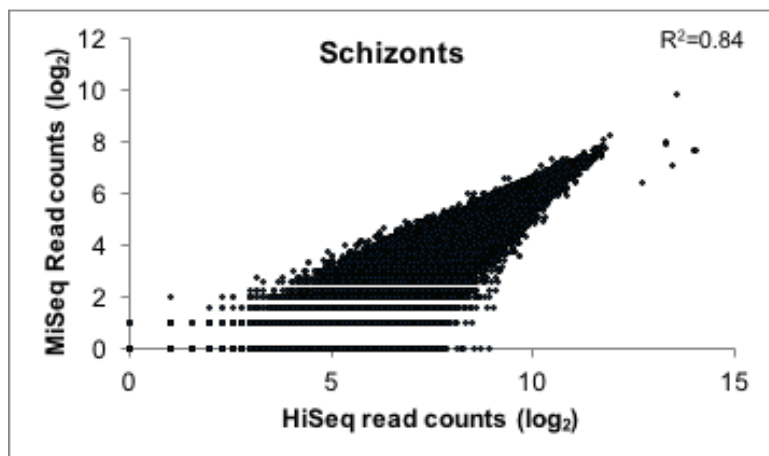

b

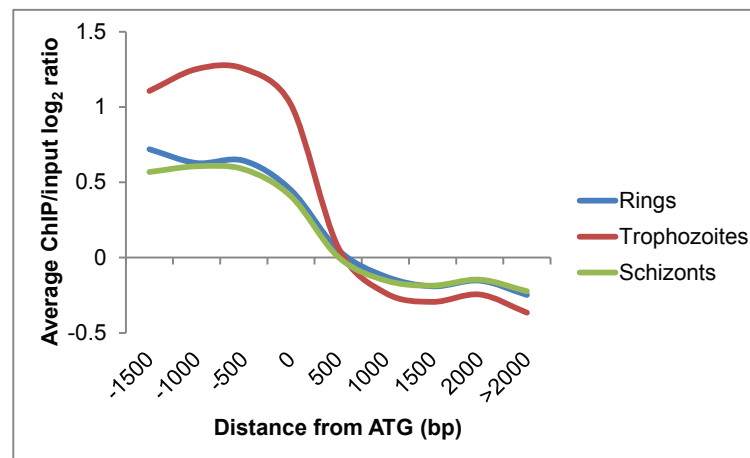

Supplement: Supplementary file 9 — Additional file 9. Figure S4. ChIP coupled to high throughput sequencing (related to Fig. 4). [file 13072_2017_147_MOESM9_ESM.pdf]
